# Tracing Europe-Wide Copper and Bronze flow in the Dutch Late Neolithic to the Early Iron Age

**DOI:** 10.1371/journal.pone.0348751

**Published:** 2026-09-08

**Authors:** Stephen William Merkel, Stijn Arnoldussen, Liesbeth Theunissen, Bertil van Os, Judith van der Leije, Joris Brattinga, Luc Amkreutz, Bibi Beekman, Irini Biezeveld, R. Alan Williams

**Affiliations:** 1 Department of Earth Sciences, Vrije Universiteit, Amsterdam, The Netherlands; 2 Institute of Archaeological Studies, Ruhr Universität, Bochum, Germany; 3 Groningen Institute for Archaeology, Rijksuniversiteit Groningen, Groningen, The Netherlands; 4 Cultural Heritage Agency of the Netherlands, Amersfoort, The Netherlands; 5 Archol B.V., Leiden, The Netherlands; 6 National Museum of Antiquities, Leiden, The Netherlands; 7 Limburgs Museum, Venlo, The Netherlands; 8 Drents Museum, Assen, The Netherlands; 9 Department of Archaeology, Durham University, Durham, United Kingdom; Instititut Català de Paleoecologia Humana i Evolució Social (IPHES-CERCA), SPAIN

## Abstract

The study of the production, movement, processing and consumption of copper and bronze objects in the European Bronze Age (*c.* 2500–800 BC) reveals the underlying mobility and interconnectedness of Bronze Age societies. Long-distance exchange networks spanned the European Continent, and this paper fills an important geographic gap in Bronze Age metal research improving our understanding of the metal flows across Europe. The Netherlands as maritime/riverine east-west and north-south crossroad is a key piece to the Bronze Age metal supply puzzle. More than 200 lead isotope and elemental analyses of copper and bronzes from the Netherlands, featuring the strategic use of portable laser ablation, constitute a key opportunity to test and reshape theories on Bronze Age metal flow. Our analyses indicate that the earliest copper is of Balkan and Slovakian origin with some import of Iberian arsenical alloys followed by an intensification of Slovakian/Únětice metal imports during the Early Bronze Age. In the early phase of the Middle Bronze Age, the Netherlands received fresh copper with nickel and arsenic impurities from both the Great Orme mine in Wales and Mitterberg in Austria, shifting towards Italian Alpine copper in the late Middle Bronze Age. Italian Alpine copper was widely used, probably reaching the British Isles via trade routes through the Netherlands. In the Late Bronze Age, high-impurity antimony-bearing copper sources prevailed in both the Netherlands and in Britain and highly leaded bronze became commonplace, with the added lead being consistent with British sources. The high-impurity element pattern can be linked to recycled Alpine metal with almost no visible contribution of Iberian metal arriving via Atlantic trade routes.

## 1 Introduction

The production, movement, processing and consumption of copper and bronze objects in the European Bronze Age (*c.* 2500–800 BC) has received a great deal of attention over the past decade [1–5]. The results illustrate how Bronze Age societies were interconnected, and how such networks of contacts shifted over time. The studies of ores and artefacts have shown that exchange networks functioned on a vast European scale, including the Mediterranean and possibly beyond. Until now, the Netherlands have played only a minor role in this very extensive body of research and discussion. Although some metallurgical studies were published in past decades, these were focused on specific types of objects, such as the copper daggers from Bell Beaker graves [6] or iconic museum pieces [7] or spectacular hoards such as the Wageningen [6,8] and Ommerschans depositions [9]. Therefore, the role of the Netherlands as a hub in various prehistoric exchange networks is in great need of assessment.

From a north-western European perspective, the Netherlands is often considered a blank spot or periphery in overviews of metal routes and exchange networks of the Bronze Age [10–16]. However, for the various phases of the Bronze Age, connections were evident [17]. The oldest copper artefacts reliably documented for the Netherlands so far are the awls and tanged dagger blades of the Late Bell Beaker period, whose elemental compositions hint at both Atlantic and south German contacts [18]. For the final Neolithic and Early Dutch Bronze Age (c. 2200–1800 BC), incorporation into a North Sea-Channel maritory [10] is evidenced by specific insular axe types [19–21] or faience beads [22,23] and pots crafted in styles shared across the maritory [24–26]. For the Middle Bronze Age (c. 1800–1100 BC), the Netherlands are reconstructed to be situated at the interface of Atlantic, Nordic and Tumulus Culture exchange systems [11,13–15,27–29]. The only three previously published lead isotope analyses from Dutch Bronze Age objects are from the MBA Voorhout hoard, demonstrating ties to the Great Orme mine in Wales [30]. For the Late Bronze Age (c. 1100–800 BC), no consensus exists on what the dominant exchange relations were: some authors suggest Atlantic [31–33] or Urnfield/Alpine [27,34,35] dominance, whereas others situate the area at the transition between these two [27,36–40] or see the area as devoid of contacts [32,41–43].

Despite the abundance of interpretations of exchange relations for the Netherlands across the Bronze Age, the metal itself has paradoxically played a minor part in such discussions. Furthermore, from a macro-geographic perspective, the Netherlands’ role as a metal transit zone for long distance networks transecting Europe is almost entirely unexplored. To fill this gap in our understanding of Bronze Age metal networks, 201 objects from the Low Countries were investigated using lead isotope analysis and, when possible, high-resolution elemental analysis to explore the changing flows of copper and bronze from the fourth millennium BC to the Early Iron Age (ca. 600 BC). The study’s main research goals are to identify potential metal sources, the frequency of recycling and the direction of metal flows within and via the Netherlands with the ultimate aim of enhancing knowledge of metal supply patterns across Bronze Age Europe.

## 2 Materials and methods

### 2.1 Archaeological objects

We selected 184 copper alloy artefacts from the 3300 objects registered in the Netherlands Bronze Age Catalogue (NBAC) and 17 objects found in Flanders (Belgium) (Fig A in S1 Appendix). The main criteria were a representative selection of functional categories, a balanced temporal distribution across the various phases (from pre-2200–600 BC), a wide geographic scope and a reliable archaeological context. Among these objects, 91 objects are from three major Dutch museums, followed by 53 objects managed by provincial repositories. A total of 20 private owners (all avid metal detectorists) were contacted, and this resulted in 29 additional artefacts. A total of 19 objects originated from municipal repositories, five from university collections and four from other heritage organisations. In terms of object categories, axes (n = 70), swords (n = 25) and spearheads (n = 23) are best represented (n > 10), but twelve other object groups are represented by at least three items (Fig B in S1 Appendix). No evident copper or bronze ingots are currently known from the Netherlands, and these therefore are not part of the study. Also, evidence of metalworking in the Netherlands is scarce. We included two bronze moulds (DB 643/DB 1124) both dredged from river Meuse, and three casting jets from the Oost-Maarland hoard [44].

**Fig 1:**
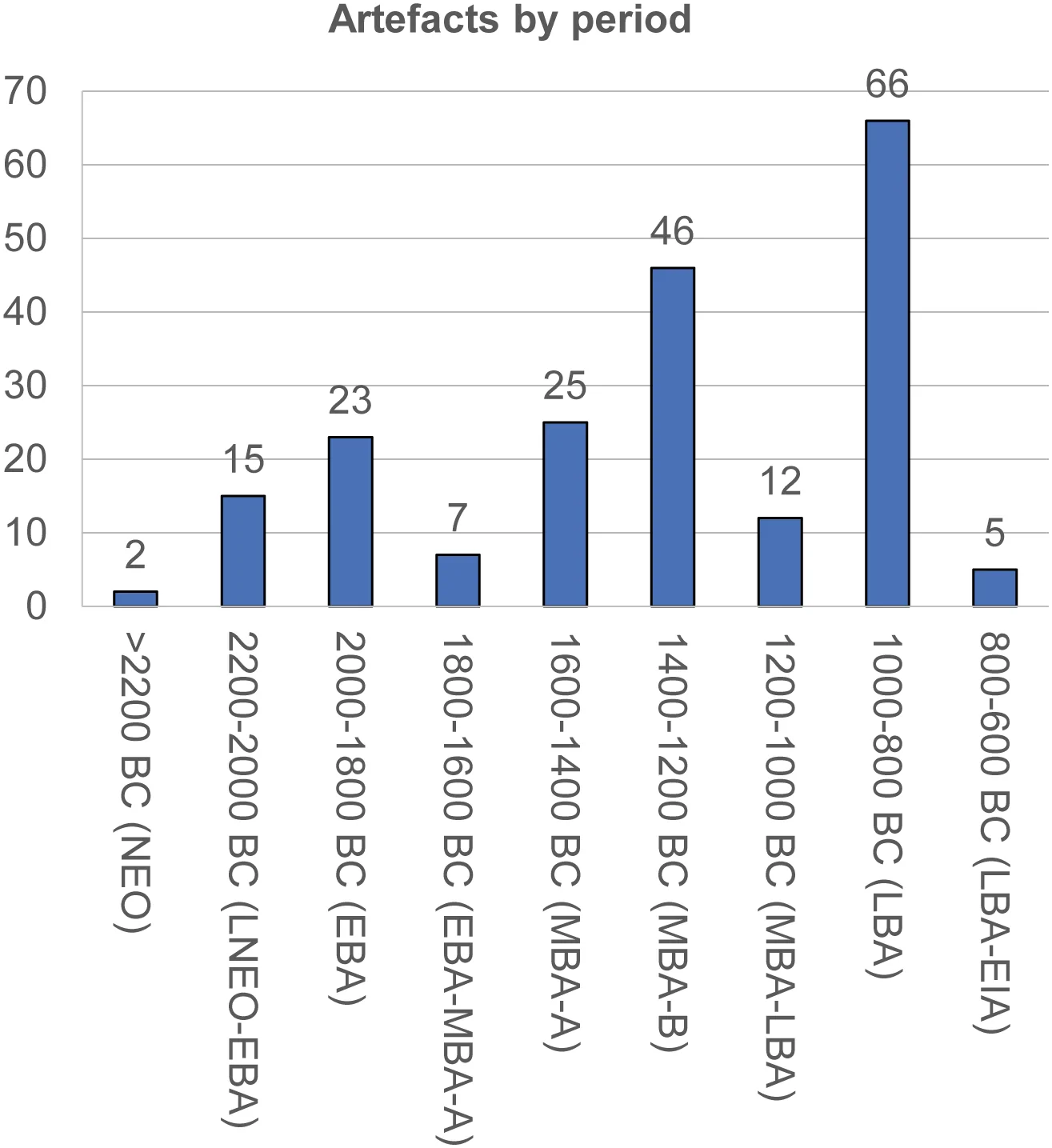
Chronological distribution of investigated objects (diagram: S. Arnoldussen, S. W. Merkel).

Chronologically, the artefacts are spread across all periods, but the distribution reflects the relative growth in the number of Bronze Age artefacts over time, with the greatest portion of objects dating to the Middle (1800−1000 BC) and Late Bronze Age (1000−800 BC) (Fig 1). The Late Bronze Age, with 66 objects, constitute the largest group and thus may create some temporal bias. Whereas most objects represent isolated finds, for various periods hoard assemblages could be studied: the LNEO-EBA Wageningen hoard [6,8], the MBA hoard of Ommerschans [9], additional palstaves from the Voorhout hoard and various LBA hoards comprising weapons, tools and ornaments (Oost-Maarland [44]*;* Nederviersel-Pulle (Belgium) [45]*;* Markerwaardweg [46]. The hoards of Borger-Drouwenerstraat [48] and Heiloo [49] represent the transitional LBA-EIA phase.

### 2.2 Theoretical approach

The combination of lead isotope analysis and quantitative elemental analysis for source identification and to explore metal flow has a long history, and the theoretical basis is well established [50,51]. In this study, the first step is to use high-quality lead isotope analyses to confirm or rule out potential metal sources and/or recycled source materials and to establish or refute relationships between metalwork assemblages across space and time. The second step is to use elemental analysis in conjunction with lead isotope data to confirm the consistency of alloys and trace element patterns with potential source materials and to inform the interpretation. The elements Ag, Ni, As and Sb are given particular weight in the interpretation; though it is known that they do not remain static throughout metallurgical processes and recycling [52,53], they have proven to be effective in differentiating sources and ore types and revealing spatial and chronological trends [50,51]. We use a level of 0.12% wt. normalized to copper as a threshold for classifying and describing impurities, but we identify trends that cross this threshold, mindful of its arbitrary nature. Lead isotope ratios combined with elemental compositions are interpreted using a large compilation of ore and object data integrating open-access databases such as IBERLID [54] and OXALID [55] with additional published studies. We attempt to interpret the data we obtained through lenses informed by holistic theoretical frameworks that integrate concepts of provenance, metal flow and recycling [56].

### 2.3 Analytical methods

This study uses a combination of non-destructive and minimally destructive techniques to obtain elemental compositions and lead isotope ratios. Fifty-five particularly fragile and/or rare museum objects were analysed using non-invasive techniques at the museums: portable X-ray fluorescence (pXRF) and portable laser ablation (pLA) to obtain samples for lead isotope analysis. The pXRF methodology is described in [57]. In brief, both a Thermo Fisher Niton Xl3t GOLD++ and an Xl5 + were used for the analyses of the surface of the objects and the drilled sample. The measuring mode used for both instruments was the *electronic mode*, and the machines are factory calibrated with several reference materials for all reported elements. Measuring time per spot was 30 s. The objects were measured six times at surface locations with least visible corrosion or iron hydroxide layers whereas the drilled samples were measured twice through pure cellulose ultrathin weighing paper (Fisherbrand™ Low-Nitrogen Weighing Paper) with the pXRF mounted in a closed lead-shielded stand under laboratory conditions. Further details on data processing and data quality can be found in S2 Appendix.

The pLA sampling for lead isotope analysis was used following previously described methods [58,59]. Once the approximate lead concentrations are known, the objects were ablated using a spot size of 100 micrometres in inconspicuous locations to collect approximately 1 microgram of lead. Discrete areas with thin or no patina were prioritized. The number of ablations needed is proportional to the lead concentration of the metal and ranged between 2–20 ablations per object. Detailed before and after photographs were made noting the sampling locations, which are otherwise not visible to the untrained, unaided eye. Multiple environmental/methodological ‘blanks’ were collected during sampling to estimate the level of contamination to be expected. These ‘blanks’ were collected by pumping air for 10 minutes; the contamination reflects the cleanliness of the sampling equipment and environment.

For solution-based mass spectrometry, we obtained drilled samples for 146 objects, which included some samples previously taken by Butler in the 1960/70s. Samples were collected using 1 mm stainless steel drill bits in inconspicuous locations or within the drill holes from older studies. Sample locations were recorded and, depending on curatorial protocols, closed with coloured wax. A maximum of 10 milligrams was needed for all analyses and excess sample material is stored for future use. Sample sizes used for analysis were typically in the order of 7.5 milligrams of fresh metal (inner quartile range: 4.2-9.8 mg).

Two objects were selected for both pLA and two independent drillings to allow direct comparison of the Pb-isotope results of the two sampling methods as well as two separately drilled and processed samples from the same object.

All drilled samples were first analysed by pXRF before further processing. The samples were weighed and dissolved in dilute aqua regia (1:6 14M HNO_3_: 6.5M HCl) in Savillex™ PFA beakers on a hotplate (105°C) overnight. This was dried down and taken up in 6.5M HCl to make a 1000 ppm stock solution. Aliquots from these stocks were taken for elemental and lead isotope analysis. Elemental analysis was performed using an iCAP™ TQ ICP-MS at the department of Earth Sciences at the VU Amsterdam. The calibration stock solution was made from single-element standards to cover the range of expected compositions. Seven copper-alloy reference materials (6x CHARM; 1x NIST) were processed using the same methods as the samples and measured alongside the samples. The concentrations of 18 elements were measured (Cu, Pb, Sn, Ag, Ni, As, Sb, Zn, P, Cr, Mn, Fe, Co, Se, Cd, Te, Au Bi), six of which (P, Mn, Fe, Ni, As and Se) were measured in KED mode using helium to lower isobaric interferences. Analytical totals were calculated and the data are normalized to 100% to facilitate comparability.

To prepare for lead isotope analysis, aliquots from the sample stock solutions containing 1 microgram of lead were dried down in separate Savillex™ PFA beakers. A bronze reference material from the CHARM set was processed along the samples. These, the pLA samples and the pLA/method ‘blanks’ were processed using HBr-HCl anion-exchange chromatography to purify the lead fraction [58]. The purified lead fraction was then diluted to 25ppb and analysed by multicollector inductively coupled-plasma mass-spectrometry (MC-ICP-MS) using a Thermo Neptune™ or a Thermo Neoma™ at the VU in Amsterdam. The lead isotope ratios were normalized using the standard bracketing technique to the NIST981 values of [60] and analyses with the Neoma were additionally doped with thallium spikes for additional mass fractionation correction. A quality control standard (CPI) was run every 5–7 samples to assess the accuracy and precision of each session.

Detailed presentation and discussion of standards and data quality can be found in the supporting information: Supplement document S2 Appendix for data quality discussion and S3 and S4 Tables for analyses of elemental and Pb isotope standards. The full dataset can be found in S5 Table, which includes a full list of the objects investigated in this study, their find location and complete repository information, including museum name and geographic location. Locality information is provided as legally allowable and further information will be provided upon request to qualified researchers with permission from the private persons involved. Access and sampling permission was granted by all institutions and private individuals involved in this study

## 3 Results

### 3.1 Earliest copper and bronzes (pre-2200 and 2200−1800 BC)

#### 3.1.1 Late Neolithic (Pre-2200 BC).

**Fig 2:**
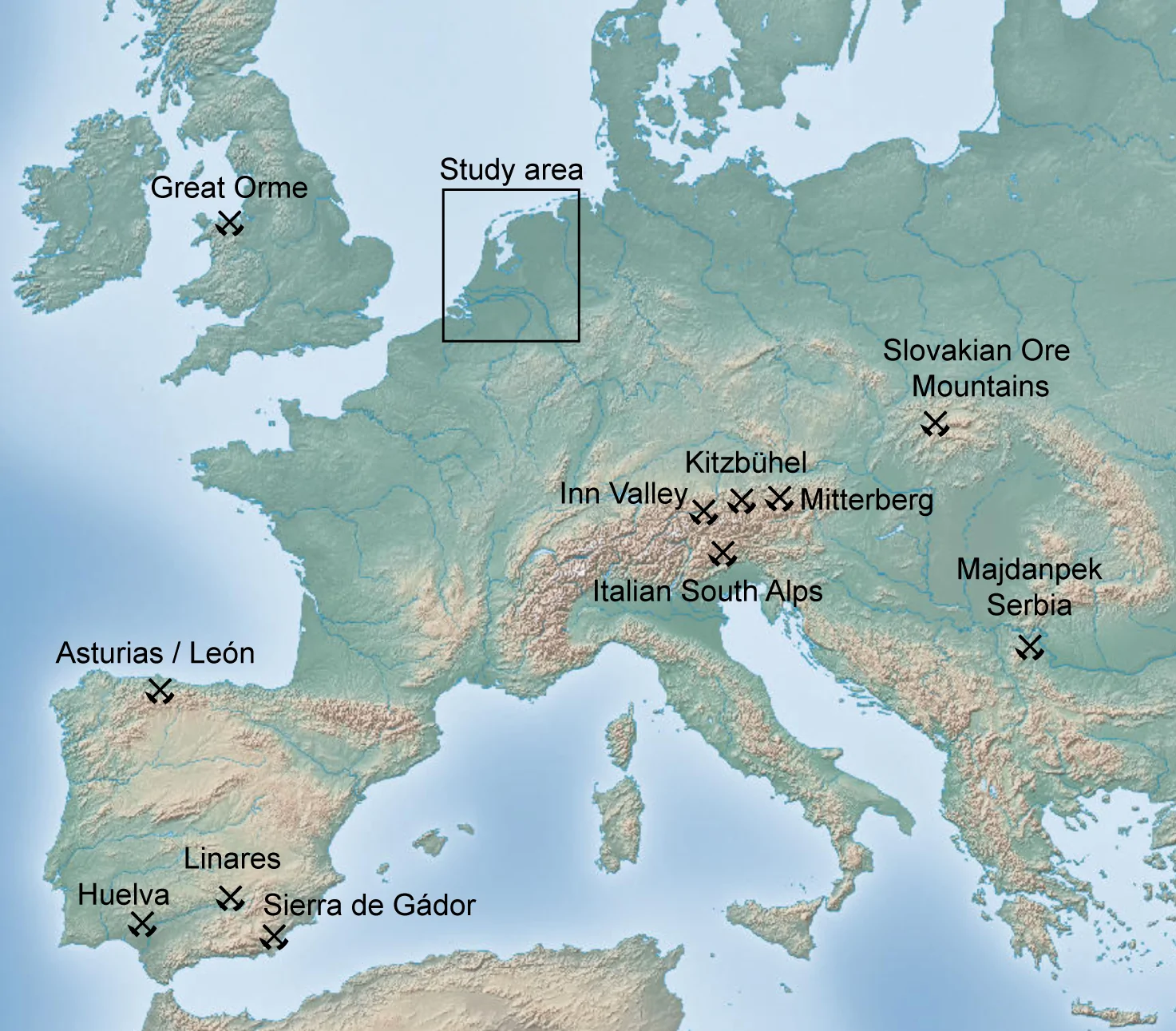
Map showing the location of copper ore deposits discussed in the text. The find locations of metal objects investigated in this study are marked by blue dots (map: S. W. Merkel). Base map © Esri. Sources: Esri, TomTom, Garmin, USGS, FAO, NOAA. Map image is the intellectual property of Esri and is used herein under license. Copyright © 2026 Esri and its licensors. All rights reserved. Reprinted under CC BY license with permission from ESRI (https://doc.arcgis.com/en/arcgis-online/reference/static-maps.htm).

The two earliest Late Neolithic objects in our study are axes with stylistic affinities to axes of Central Europe. The flat axe / axiform ingot (DB 1352) from Glanerbrug is stylistically attributed to the Central European Early-Middle Chalcolithic (4^th^ millennium BC) and is related to axes spread from the Balkans to the Baltic [61]. It is made of unalloyed copper with traces of silver (1400 ppm), antimony (970 ppm) and arsenic (700 ppm) and very low nickel and lead (< 50 ppm). The lead isotope ratios are not consistent with the broader Slovakian ore field but are particularly comparable to Neolithic-EBA Serbian/West Balkan artefacts and ore from the copper mine of Majdanpek in Serbia (Fig 2; Fig C in S1 Appendix) [62–64]. Flat axes with similar lead isotope ratios and elemental composition from Germany and Southern Scandinavia are interpreted as coming from Serbia [65]. While the relatively high levels of As, Ag and Sb are just within the range of Chalcolithic Serbian/West Balkan artefacts (Fig D in S1 Appendix) [62,64], these are inconsistent with the lower trace element levels of the few analyses of native copper and malachite from this deposit [62]. However, the handful of analyses probably do not adequately reflect the full range of ore compositions from this enormous copper deposit [66].

The Zabitz-Westeregeln-type double axe, (DB 613 [61]) from Escharen is made from an “arsenic only” arsenical copper (0.8%). It has elevated gold (21 ppm) and the highest tellurium (154 ppm) compared to any other BA objects from our study. The lead isotope ratios are geologically young and compare well with ore and artefacts from Slovakia from the late 4^th^/3^rd^ millennium BC (Fig E in S1 Appendix) [67–69] and contemporary artefacts interpreted as being made from Slovakian copper [65]. The lead isotope ratios of this axe are consistent with the Schreiner’s Slovakian artifact cluster 5, which is mainly composed of middle Chalcolithic to EBA arsenical copper objects [69]. This double axe is elementally and isotopically analogous to the arsenical copper Zabitz-Westeregeln-type double axes found in northern and western Germany [65], interpreted as being made from Slovakian copper. Our example was presumably produced in central Germany [61], from metal produced from Slovakian ores.

#### 3.1.2 Late Neolithic/Early Bronze Age (2200−1800 BC).

**Fig 3:**
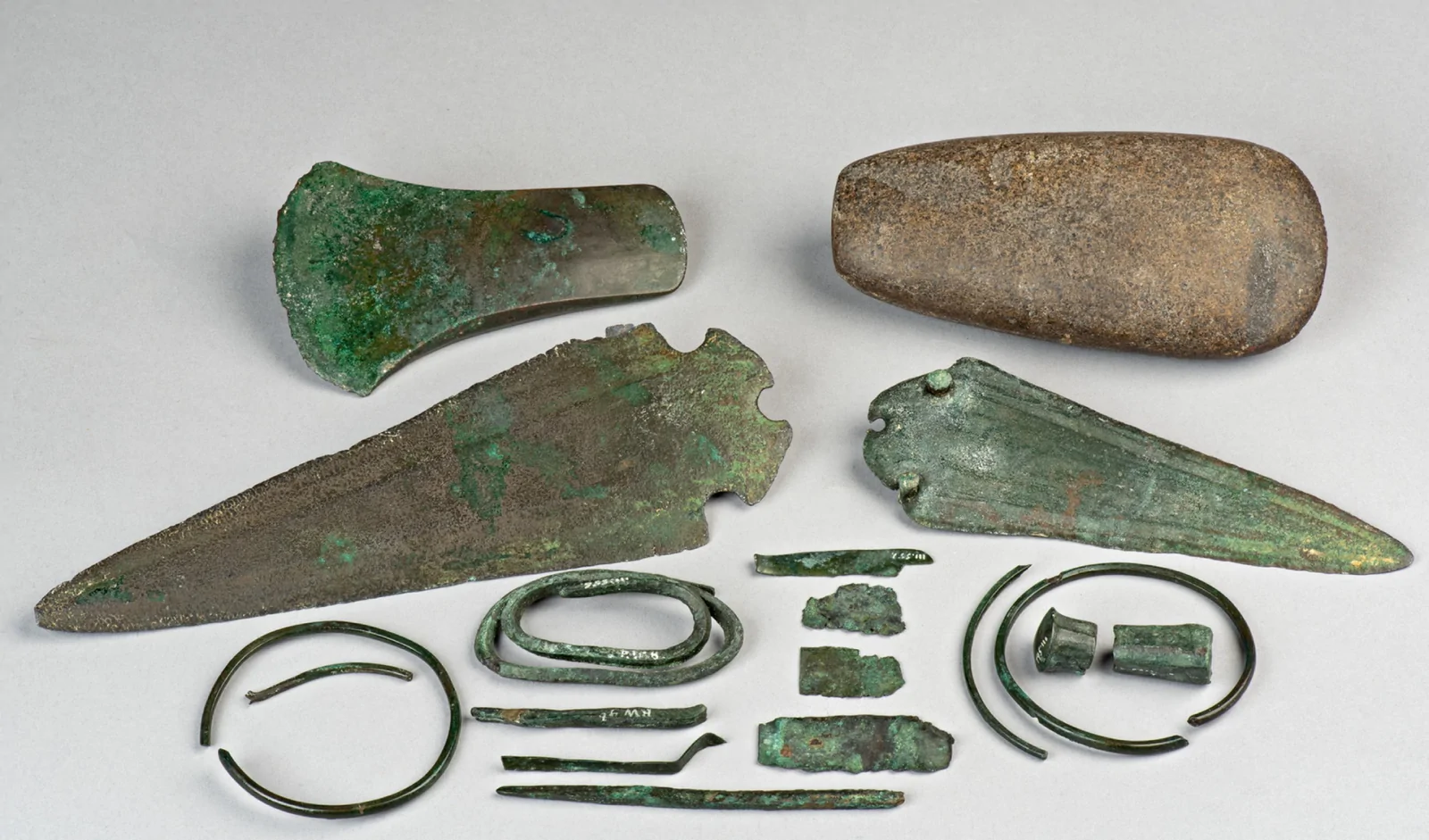
The Late Neolithic/Early Bronze Age Wageningen hoard. Reprinted under a CC BY license, with permission from Rijksmuseum van Oudheden, original copyright 2003.

Here, we discuss objects datable to the final centuries of the Late Neolithic B (2500−2000 BC), and Early Bronze Age (2000−1800 BC) in the Dutch chronology. Most notably, the Wageningen hoard (Fig 3) is placed at the transition of LNEO-B to EBA (ca. 2200−2000 BC).

In the earliest phases of the Chalcolithic, there have been Bell Beaker Culture Atlantic connections bringing metals from Iberia to the British Isles [70]. In the Chalcolithic and Early Bronze Age, the Netherlands is described as having connections to that Bell-Beaker “Atlantic” and the continental European Únětice Culture [6,18]. In the Netherlands, the patterns in copper composition from this period appear to reflect these connections, both from high impurity fahlore copper with parallels to the Únětice Culture and arsenical alloys referred to in the past as “Dutch Bell Beaker” metal [6] or simply “Bell Beaker” metal [71]. These two alloy types are also identified in our analyses for this period, although the separation of the groups is not complete. There is a strong tendency for alloys with greater than 2000 ppm Sb to have elevated Ni, Ag and As, while alloys with less than 1000 ppm Sb are also poor in Ni and Ag. Therefore, Sb was used as the primary discriminator for dividing fahlore and arsenical alloys.

**Fig 4:**
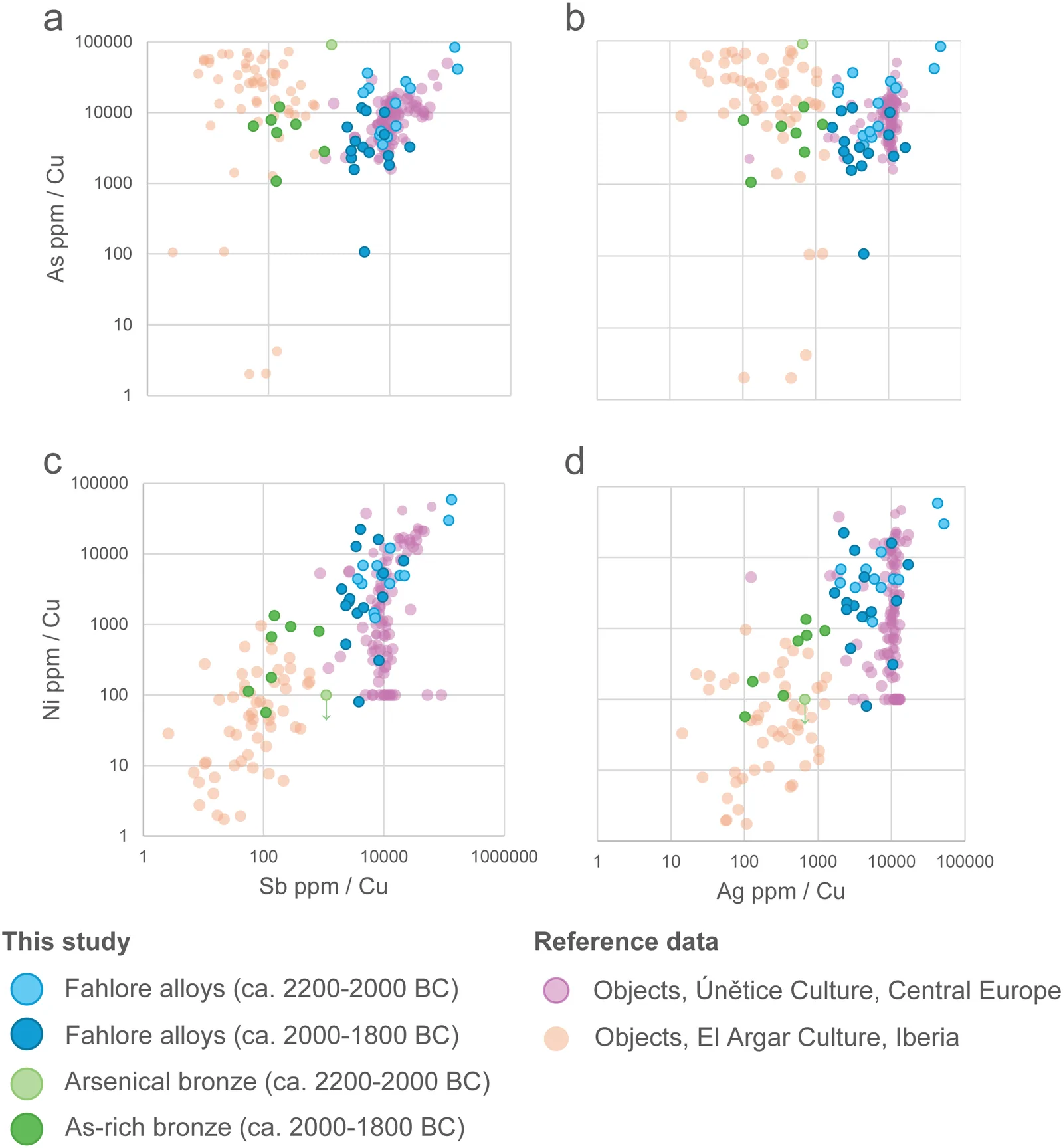
a-d) Elemental ratios of Early Bronze Age alloys in the Netherlands (pXRF and ICPMS) compared to EDXRF analyses of Únětice metalwork in Bohemia/Saxony [73] and Saxon-Anhalt (Ni-fahlore groups only, [72]) and ICP-MS analyses of Argaric metalwork in Southeast Spain [87–90] (diagrams: S. W. Merkel).

**Fig 5:**
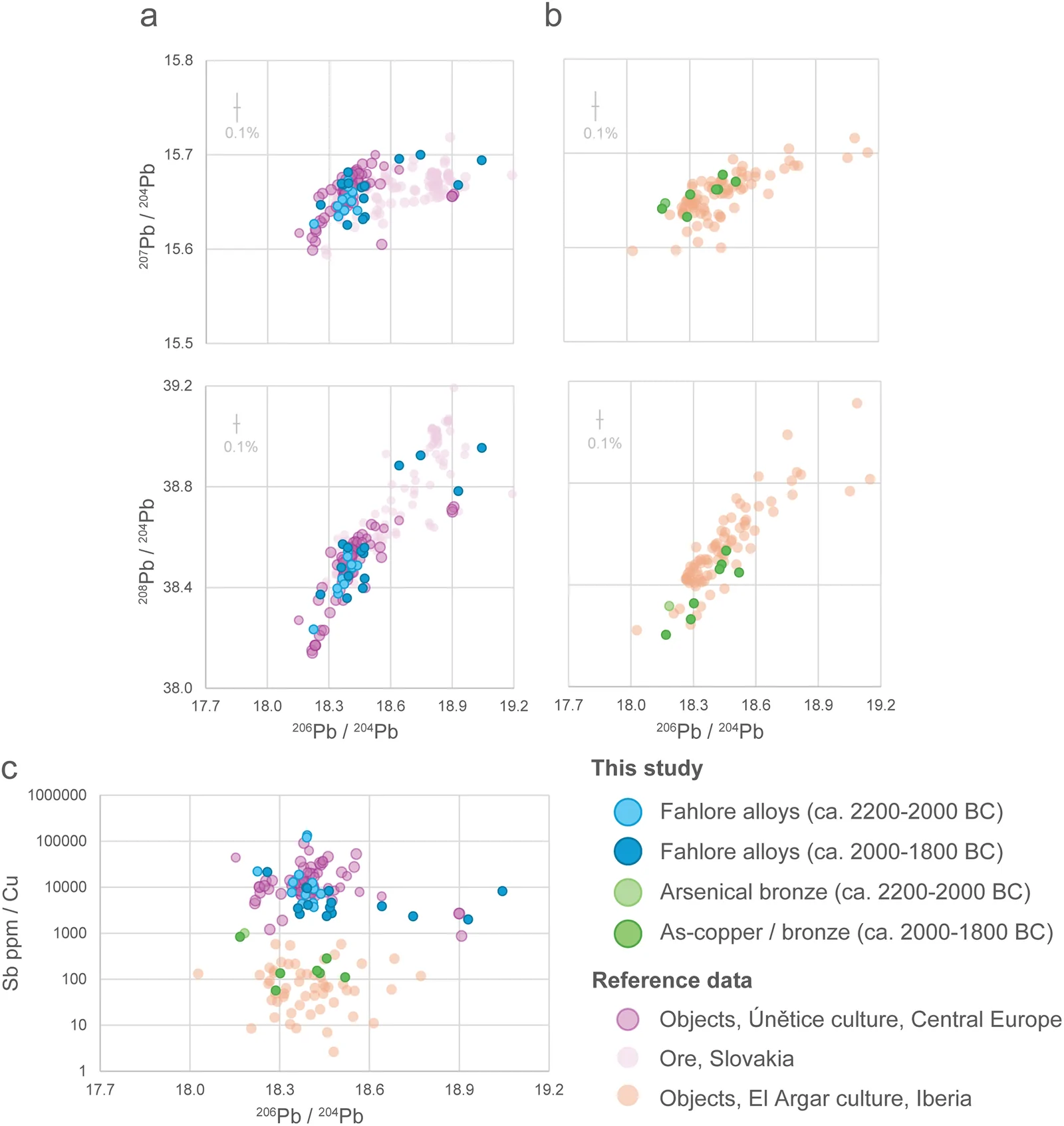
a) Lead isotope ratios of Early Bronze Age fahlore alloys in the Netherlands compared to analyses of Únětice metalwork in Bohemia/Saxony [73] and Saxon-Anhalt (Ni-fahlore groups only, [72]), b) lead isotope ratios of Early Bronze Age arsenic alloys in the Netherlands compared to analyses of Iberian and Argaric metalwork [87–91], c) lead isotope ratios vs. Sb/Cu of EBA objects in the Netherlands (pXRF and ICPMS), Únětice metalwork [72,73] and Argaric metalwork (ICP-MS only, [87–90]) (diagrams: S. W. Merkel).

Nickel-bearing fahlore copper alloys containing Ag, Ni, As and Sb above trace amounts (>1000 ppm, typically above 2000 ppm to low wt. %; i.e., Singen metal) are prominent in the Wageningen hoard (ca. 2200–2000 BC) (Fig 3) and are common among finds dated to the EBA (2000–1800 BC). While tin is usually present in the percentage range in the latter part of the EBA, in the Wageningen hoard only half of the objects indicate tin concentrations above 1 wt. %. The objects that have lower tin contents are the awl, sheet metal fragments and rivets. The Wageningen halberd rivets have high arsenic, nickel, antimony and silver impurities, while the other Singen-type compositions typically have lower concentrations of these elements. The lead isotope ratios and the compositions are consistent with nickel-bearing fahlore Singen-type alloys used by the Únětice culture in Bohemia/Saxony and Saxon-Anhalt (Figs 4 and 5) [72,73] and ore from Slovakia [67–69]. Nickel-bearing fahlore compositions are common in Saxony, and two-thirds of the 28 EBA objects analysed in Slovakia have similar elemental proportions [69], so it is certainly possible that alloying and remelting within the Únětice cultural sphere could have partially homogenized the elemental pattern, narrowing the elemental and isotopic range. And although the distinctive Ösenring-type fahlore elemental patterns with low nickel and lead concentrations (i.e., C2 copper: fahlore with no/traces of Ni [74]) are found in the Únětice cultural area (e.g., [73]), this elemental pattern is not clearly identifiable in our dataset, again possibly lost through mixing.

Our analyses confirm the presence of other copper sources, chiefly arsenical coppers and arsenical bronzes with low levels of Sb, Ag and Ni between 2200 and 1800 BC. Historically, these alloys have been interpreted as being connected to the EBA Bell Beaker culture spread over the European Atlantic [71].

**Fig 6:**
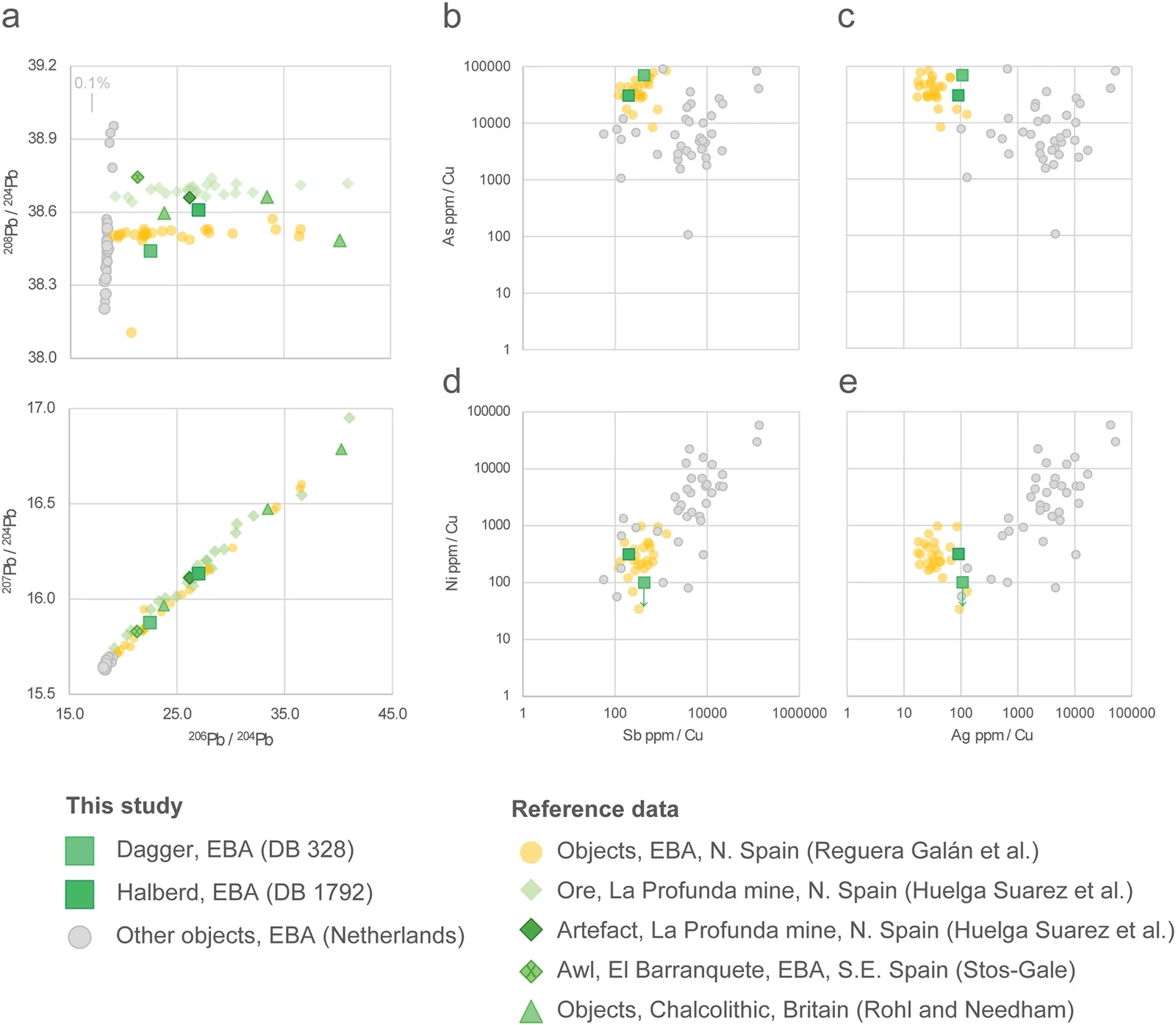
a) Lead isotope ratios of radiogenic arsenical bronze artefacts: EBA dagger from Wageningen and halberd from Roermond, compared with Chalcolithic arsenical alloys from Northern Spain [78], copper ore from the La Profunda mine [77], arsenical copper awl El Argar [76] and radiogenic Chalcolithic arsenical artefacts from the British Isles [75]. b-e) elemental ratios of highly radiogenic EBA arsenical bronzes found in the Netherlands (pXRF/ICPMS) compared to published ICPMS trace element data from Chalcolithic Northern Spain [77,78] (diagrams: S. W. Merkel).

Objects sampled in the 1990s from Britain’s oldest Chalcolithic phase were shown to have extremely radiogenic lead isotope ratios and the source could not be identified at the time, but it is likely that this copper came from the Asturian-Leonese mines in Northern Spain [70]. Three of these British objects, all arsenic alloys (halberd, dagger, flat axe), are plotted in Fig 6 [75]. A further analysis of an arsenical copper awl from the El Argar Culture has similar radiogenic lead isotope ratios (Fig 6a) [76]. In our analyses, two objects (DB 328: the Wageningen dagger and DB 1792: the Roermond halberd) show similar lead isotope ratios and are comparable to ore and metalwork from the Asturian-Leonese mines near the Cantabrian coast (Fig 6a) [77,78] All objects have very low lead contents (< 0.1 wt. %) and it should be noted that the uranogenic isotopes (^207^Pb/^204^Pb vs ^206^Pb / ^204^Pb) form a single array but that there is considerable variation in the thorogenic ratios (^208^Pb-ratios), probably due to minor contamination during ore smelting or casting. The major, minor and trace elements of these two objects (DB 328/DB 1792) are consistent with copper finds from the Asturias region near the copper mines (Fig 6b-c), making it highly probable that these are Iberian imports.

The halberd from the Wageningen hoard (DB 327) is an arsenical bronze (ca. 8 wt. % As) with geologically old lead isotope ratios. It is not comparable to Irish or Welsh copper sources [55,75,79]. Going further afield, the lead isotope ratios are close to ore from the Ossa Morena, particularly ore from the Azuaga-Fuente Obejuna [80], an area known to have been mined for copper in the Chalcolithic [81], but also similar lead isotope ratios are found in copper ore from the South Portuguese Zone [54]. The very high arsenic content indicates that this is an intentionally produced alloy that is unlikely to have been recycled, indicating it to be an Atlantic, possibly Iberian, import. Remarkably, as mentioned above, the associated rivets are made from Central European Singen-type fahlore copper, indicating the halberd blade was hafted or re-hafted after export.

In the later EBA (2000–1800 BC) phase, tin-bronzes with high levels of arsenic occur frequently. Most objects which can be stylistically attributed are locally produced axe types (flat axes and low-flanged axes) or show westward connections to Brittany and the British Isles (esp. DB 2856; possibly DB 1128) and thus attests to metal flow from the West. It has been suggested, based on the intensity of arsenical alloy use on the Iberian Peninsula, that this “arsenic-only” Bell-Beaker metal may have an Iberian source [82], though this interpretation is contested [83]. It also has been speculated that “arsenic-only” Bell-Beaker metal with tin impurities could come from Cornwall, though there is currently no substantiating mining or metallurgical evidence [84–86].

As an exercise, we compared the compositions and lead isotope ratios of this arsenic-bearing metal to EBA Argar metalwork [87–90] and Tombs of Humanejos Bell Beaker metalwork from the 2^nd^ half of the 3^rd^ Millennium BC [91]. Isotopically, they are consistent and indeed, there are strong elemental similarities with a subset of the Dutch dataset (Figs 5 and 6). Many of the objects found in the Netherlands have lower arsenic concentrations and occasionally higher Ni, Sb and Ag having values between typical Únětice Ni-Fahlore and El Argar compositions. Through the act of alloying and remelting, volatile arsenic can be lost [82], possibly explaining the generally lower arsenic concentrations of “Bell Beaker metal” while even slight contamination with Ni-fahlore during casting could be responsible for minor increases in Ni, Sb and Ag. Our data certainly supports the possibility that Iberian copper was a major contributor to the Atlantic Bell Beaker copper supply.

### 3.2 Middle Bronze Age (1800−1000 BC)

The copper-alloy compositions changed drastically in the Middle Bronze Age, reflecting the use of new Welsh and Alpine copper sources. There is almost a complete abandonment of fahlore copper in this period. A few objects, such as an early MBA A Nienborg-type low-flanged axe (DB 3285, with Ag-Sb-As-Ni impurities at 0.14-0.4% wt.) may be a carry-over from the EBA metal stocks. Fahlore copper bearing silver and antimony does not disappear completely; it is found as potential mixtures in 11–12 objects chronologically spread over the MBA and fahlore in purer form (Sb ≈ 1 wt. %) in four objects all dating to the MBA B / LBA transition. The “arsenic only” alloys used in the Early Bronze Age however fully disappear.

#### 3.2.1 Middle Bronze Age A (1800−1400 BC).

**Fig 7:**
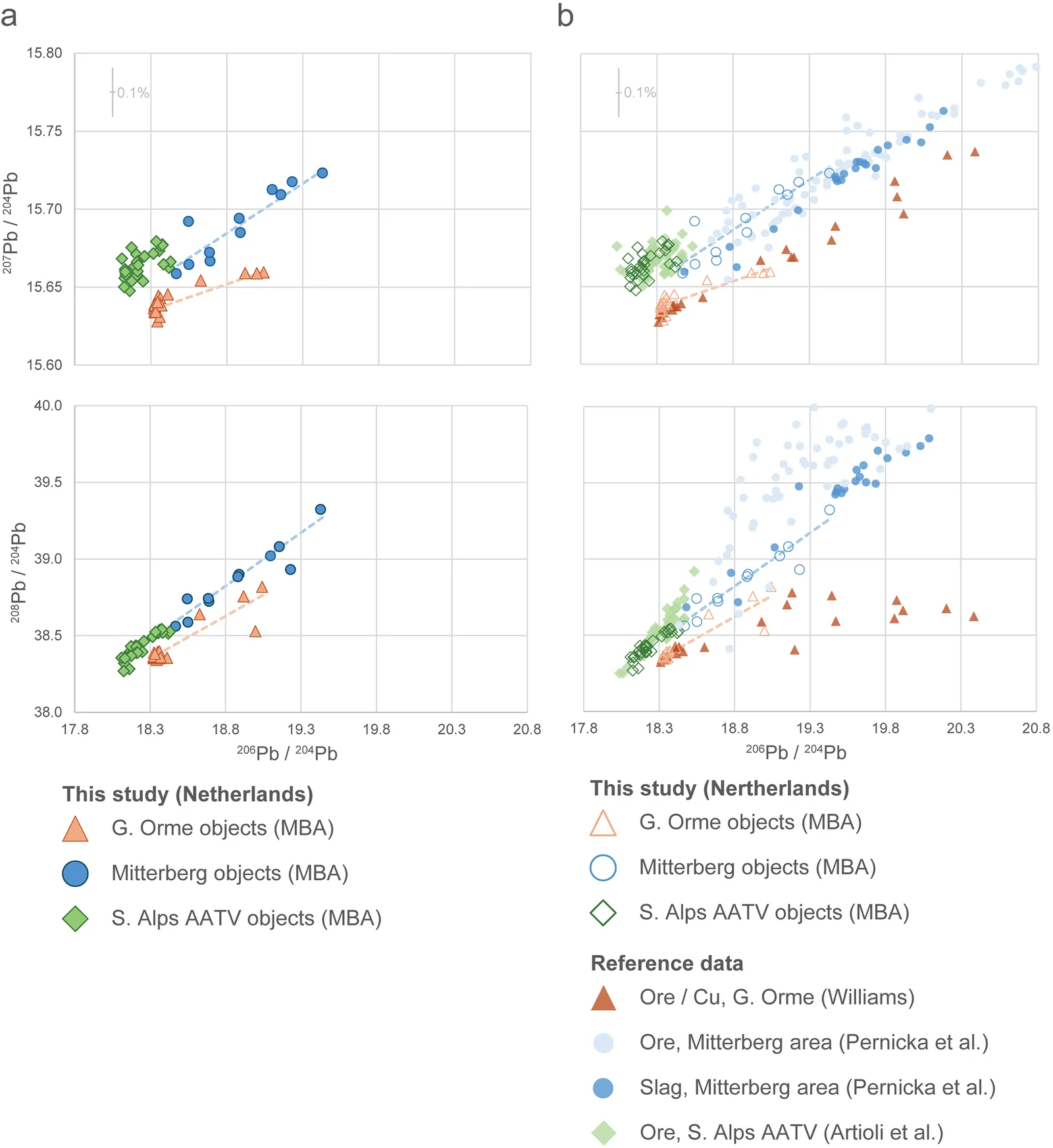
a) Lead isotope ratios of Middle Bronze Age source groups and b) ore and copper from the Great Orme [30], ore and slag from Mitterberg [95] and ore from the South Alpine AATV in Italy [103] (diagrams: S. W. Merkel).

**Fig 8:**
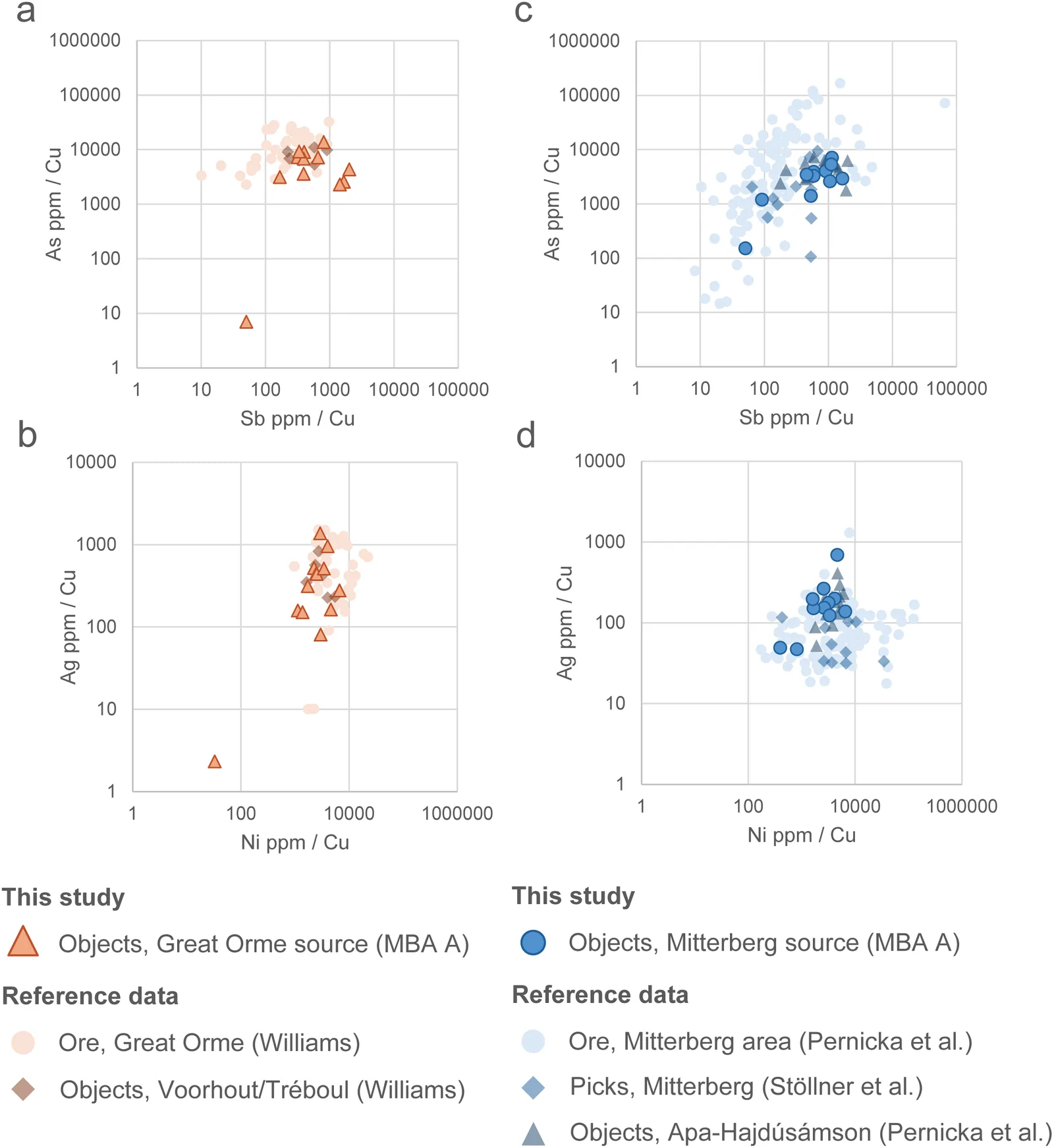
a-b) Trace element comparison of MBA A objects from the Netherlands attributed to the Great Orme source with ore and objects also attributed to the Great Orme [30] and c-d) the trace element comparison of MBA A objects attributed to Mitterberg together with reference ore and objects [95–97] (diagrams: S. W. Merkel).

The MBA A begins with a strong rise in the use of copper with arsenic-nickel impurities, and the lead isotope ratios (Fig 7) and trace element patterns (Fig 8) indicate that the copper supply was fuelled by two primary sources: the Great Orme mine in Wales and the Austrian Mitterberg.

**Fig 9:**
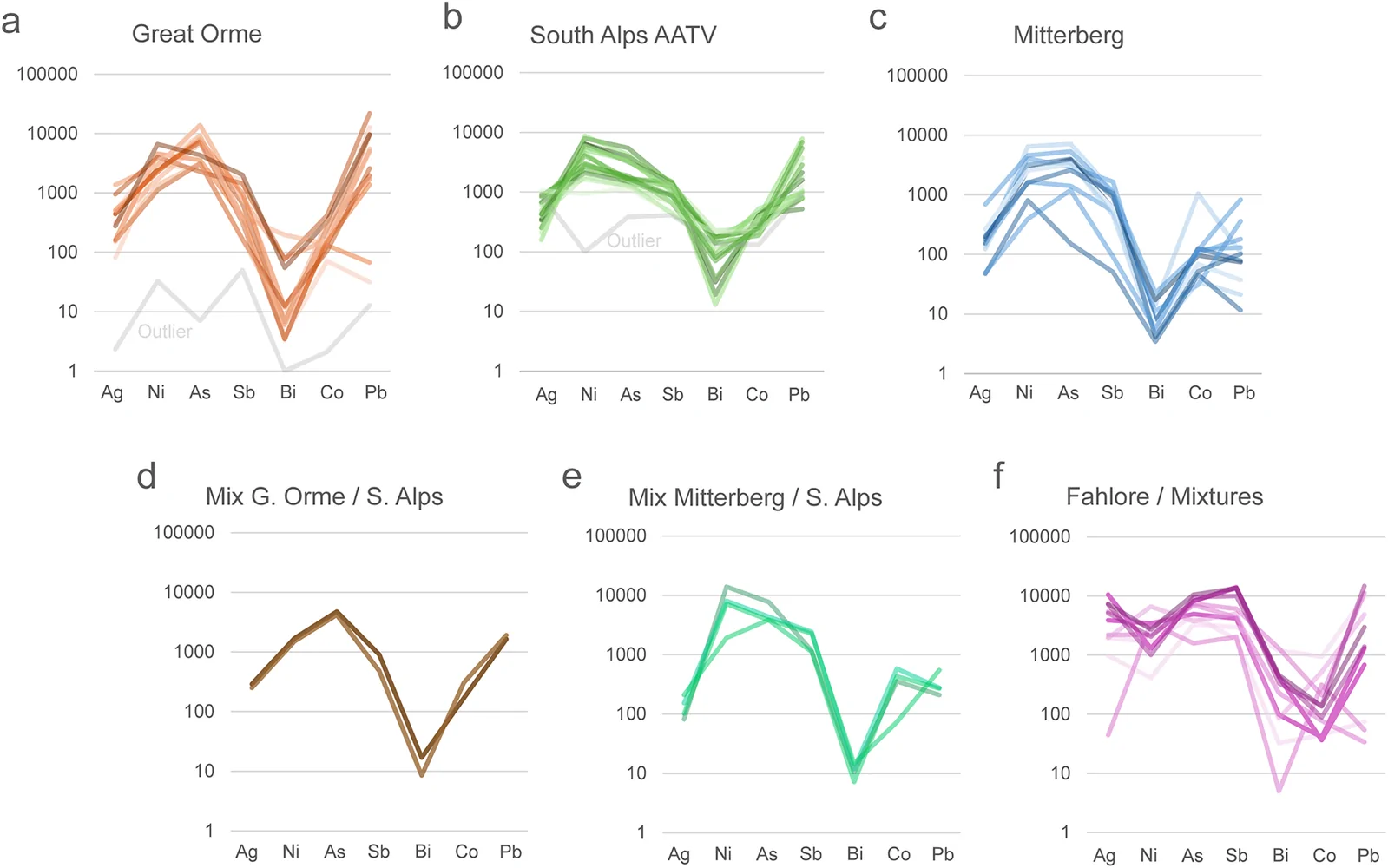
a-e) Trace element profiles of MBA objects found in the Netherlands attributed to sources and potential mixtures based on their lead isotope ratios. f) Fahlore and potential fahlore mixtures are plotted separately (diagrams: S. W. Merkel).

The dominant MBA A copper source in the Netherlands has characteristics that match copper from the Great Orme mine in Wales. The ore from the Great Orme is composed of secondary copper minerals, mainly easy-to-smelt malachite mixed with the iron-rich mineral goethite, which are all formed through the weathering of the primary sulphide ore mineral chalcopyrite [30]. The lead isotope ratios cluster on the geochemically “normal” lead signature from the mine, and the copper with low lead contents follows the radiogenic array demonstrated by low-lead ore from the Great Orme (Fig 7b) [30,92]. The elemental patterns consistently reflect arsenic-nickel impurities seen in the ore analyses which are also known to have highly variable lead contents (very low to very high) due to some of the copper veins occasionally being intersected by lead ore veins. (Figs 8a and 9a). Type Tréboul spearheads (DB 1037/DB 2993/DB 3144 [93], grooved ogival daggers (DB 122/DB 1233) and shield palstaves from the Voorhout hoard [94] testify to the strong contacts with the Atlantic zone. That being said, some imports from the south(west) German Tumulus culture area (DB 78; an axe of Type Mägerkingen [61]) or Nordic sphere of influence (DB 1652; a Wohlde sword [17]) areas show that the Great Orme supplied these areas as well.

The second major source of copper is the chalcopyrite mine of Mitterberg in the Austrian Eastern Alps where the difficulties of smelting primary chalcopyrite ores were overcome. The copper from this mine is characterized as having very low lead contents, prominent As-Ni impurities and the lead isotope ratios follow a radiogenic array with higher ^207^Pb/^204^Pb to ^206^Pb/^204^Pb ratios compared to the Great Orme (Fig 7b) [95]. The lead isotope ratios and trace elements of this group are consistent with artifacts attributed to the Mitterberg source, such as copper mining tools from the mine [96] and artefacts from the Apa hoard (Fig 8b) [97]. Regarding the lead isotope ratios of the ore, it has been noted [97,98] that there can be a misalignment on the slope of the ^208^Pb ratios of ore with the ratios of copper and slag, which is present in our data as well. One proposed explanation is that during smelting, trace amounts of lead in the smelting charge (i.e., stone, clay, fluxes) could cause shifts in the ^208^Pb ratios [97]. Because of the extremely low lead concentrations in Mitterberg copper ore and the great potential for contamination from traces of lead in stone, clay and metallurgical fluxes during smelting, it may be better to compare slag with smelted copper than with ore. The trace element pattern of copper with lead isotope ratios consistent with Mitterberg consistently has lower Bi, Co and Pb compared to other MBA copper types.

It is striking that nearly all of the objects crafted from Mitterberg ore in this phase are suspected imports: these comprise Atlantic Tréboul daggers (DB 3296/DB 3065 [93]), Nordic Sögel-Wohlde blades (DB 1609/DB 2735 [99]) a Swiss dagger blade (DB 3053 [100]) and a Type Mägerkingen axe from the south(west) German Tumulus culture area (DB 110; [61]). Moreover, a small cache of two as-cast, unfinished ‘axes’ at Emmen (DB 2054/2055) may inform us on the way the Mitterberg metal was exchanged: as rough-cast palstave shaped axiform ingots and already alloyed with tin [101].

Foreshadowing the MBA B phase, one object, a Wohlde-type sword (DB 126) [102], has lead isotope ratios consistent with South Alpine chalcopyrite-based ore from the Alto Adige, Trentino and Veneto ore fields in Italy (i.e., AATV [103]). This sword type, solidly dated to the 16th century BC, is contemporary to the very first Scandinavian axes made with South Alpine AATV copper [104].

In our dataset, although clear lead isotope mixing between source groups is exceptionally rare during the MBA A, the elemental and/or lead isotope ratios of four objects are inconsistent with the main sources mentioned. The Nienborg type axe (DB 3285), a high-flanged axe (DB 2004 [61]) and a chisel (DB 3061 [105]) all contain high enough amounts of Sb, Ni, As and Ag to suggest them to be mixtures between fahlore and chalcopyrite copper. The other, a Central European style full-handled dagger (Vollgriffdolch, DB 837 [106]), is made of an unusual tin-bronze alloy containing high levels of As (1.4 wt. %) and Ag (0.34 wt. %) but also Ni (0.24 wt. %) and Sb (ca. 0.1 wt. %). Isotopically, it is consistent with the southern Alps (AATV) but could represent an uncommon ore mixture or come from another, unidentified, source.

Where typology could be closely specified, the Great Orme group contains a proto-axe/ ingot, palstaves of Normand and Acton Park types and (non-British) “Atlantic” type high-flanged axes from the Voorhout hoard. The Mitterberg group contains two of the four Sögel-Wohlde-type swords, but one sword has lead isotope ratios consistent with Great Orme. The same is true with Tréboul-type spearheads, three of which are consistent with the Great Orme and one, Mitterberg. The two low flanged axes of Mägerkingen type are divided between the Great Orme and Mitterberg groups, and the grooved ogival daggers, two are consistent with Great Orme and the one with geometric designs, Mitterberg. This and previous work by others (e.g., [16,104,107]) stress the point that in early MBA continental Europe, the typological groups are not consistently connected to a single source material and instead reflect both the agency of metalworkers and fluctuations in the metal supply where the objects were made.

#### 3.2.2 Middle Bronze Age B (1400−1000 BC).

Large scale production existed in the Southern Alps in the Trentino, Alto Adige and Veneto ore fields, and the main phase of production was 1400–1000 BC [108–111]. The movement of this copper northward is a topic of active research. Previous work has identified a ‘metal-for-amber’ exchange system, following the route of Baltic amber into the Southern Alps at a time when copper production in the South Alpine AATV region increases and flows into the northern European copper supply [4,16,112,113]. This copper did not only reach Scandinavia.

**Fig 10:**
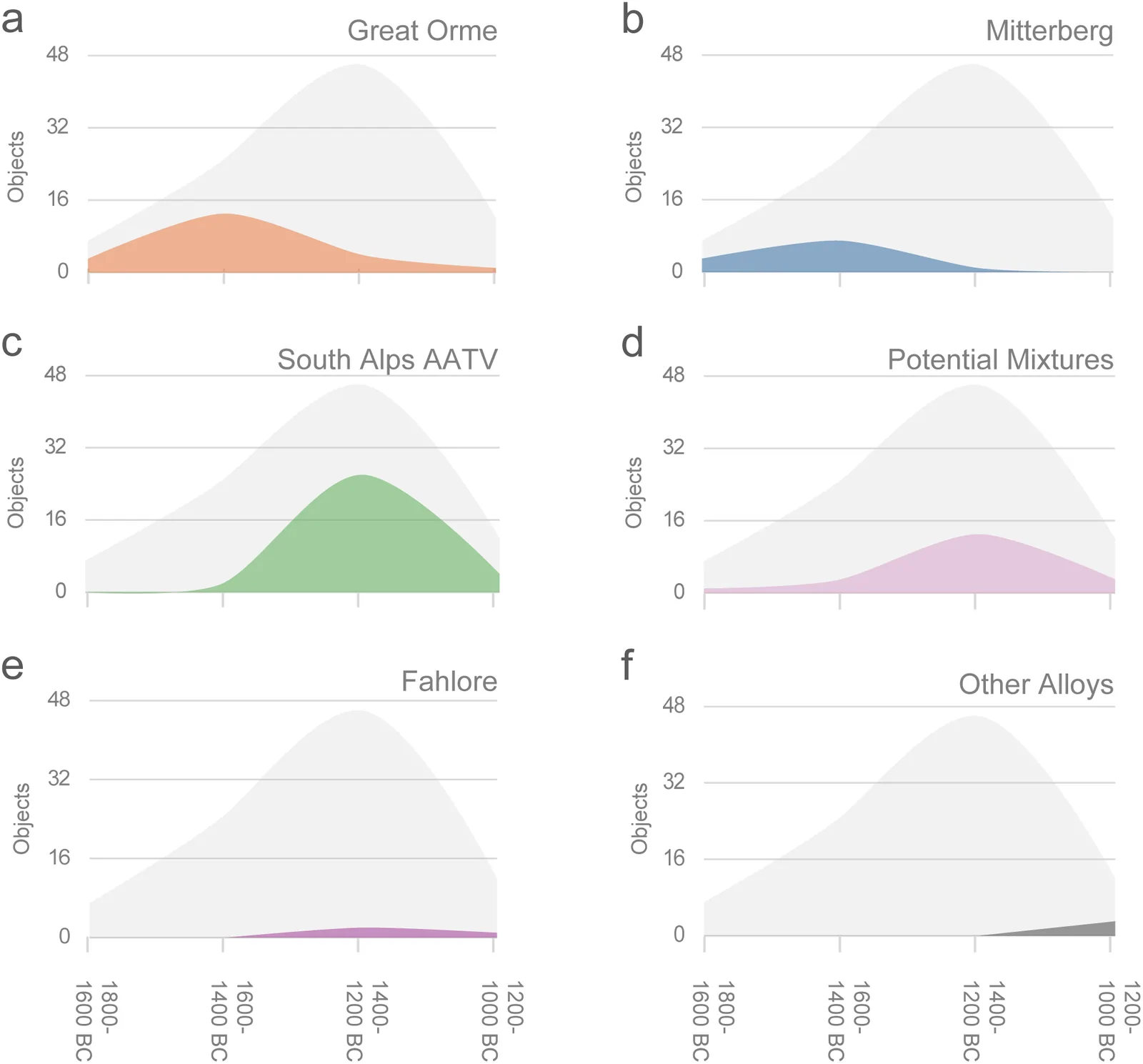
Chronological distribution of source groups across the MBA. a-c) the main chalcopyrite-type MBA groups, d) potential mixtures between chalcopyrite-types and/or fahlore, e) fahlore represented by alloys with >1% Sb / Cu and f) other alloys indicates leaded alloys or low impurity copper. (diagrams: S. W. Merkel).

The dominant group of copper in this period in the Netherlands is a Ni-As “chalcopyrite type” (Fig 9) with a relatively narrow range of lead isotope ratios consistent with the South Alpine AATV in Northern Italy (Fig 7) [103,114]. This group consists of 26 of 46 objects from the phase 1400−1200 BC and four of the twelve from 1200−1000 BC (Fig 10). Objects from the Ommerschans hoard figure prominently (n = 8), and both aggrandized dirks of the Plougrescant-Ommerschans type from Ommerschans (DB 1758 [115]) and Jutphaas (DB 1823 [9]) have AATV-type lead isotope ratios. The remainder of the AATV objects are singular palstaves (DB 64/ DB 192 / DB 156 / DB 192 / DB 1701 and DB 2769 [116]) and an Atlantic Type Rosnoën rapier dredged from the river Meuse (DB 1089 [28]).

While copper slag from Segonzano and Transacqua, to the north of Trento, dated to the Italian Recent Bronze Age (ca. 1350/1300–1000/975 BC) [117] closely match our bronzes, the full lead isotope range of Recent Bronze Age copper slag from the Southern Alps is more diverse. Most Northern Italian late MBA / LBA bronze artefacts from Friuli-Venezia Giulia plain [118] and from the Garda Lake area [119], apart from pure copper ingots, are consistent elementally and lead isotopically to the bronze found in the Netherlands from the same period (Fig F in S1 Appendix).

It is important to mention that the Southern Alps contains two main geological groups of copper ore deposits: the AATV and the Valsugana-volcanic massive sulfide (VMS) [103]. While in Italy, ore, slag and copper ingots from primary production cover the full isotopic range of the Valsugana-VMS and Alpine AATV, the range seen in bronzes is much narrower. In Scandinavia, most objects interpreted to come from the Southern Alps are consistent with the AATV and a few with the VMS [3,4], but in the Netherlands the range is even narrower and most notably the Valsugana-VMS ratios are missing. It was previously noticed with Italian swords that even in Italy, no swords match the VMS [16], and we agree that this likely reflects anthropogenic homogenization during alloying and recasting events.

Objects consistent with the Great Orme and Mitterberg are few in the period 1400−1200 BC: four and one, respectively, probably representing the tail of large-scale production earlier in the MBA. Of the Great Orme objects, two show clear Atlantic cultural affinity; a quoit-headed pin (DB 941 [120]) and a type Normand palstave (DB 3183 [121]). The blade fragment from the Vecht stream valley that was crafted from Mitterberg ore (DB 3316) could not be characterized typologically.

Two objects from the period 1400−1200 have high Sb contents (ca. 1 wt. %) together with As and Ag with lower Ni: a looped palstave (DB 2772 [122]) and winged axe (DB 3188: type Geseke-Biblis [123]). The lead isotope ratios are consistent with ore from both Slovakia and the Inn Valley [69,124].

Mismatched elemental and/or clear lead isotope mixtures between source groups are much more prevalent in the period 1400−1200 BC than in earlier periods and comprise up to 13 of 46 objects. Most of these mixtures can be interpreted to be earlier source groups mixed with S. Alpine AATV copper. This is most clear with potential Mitterberg-S. Alps (n = 4) and Great Orme-S. Alps (n = 4) mixtures that fall on potential lead isotope mixing lines (Fig G in S1 Appendix). Fahlore compositions with moderate Sb concentrations (ca. 0.4-0.6 wt. %) are scarce in this period: five objects. The lead isotope ratios suggest major lead contributions from the S. Alps (n = 3) and Slovakian and/or mixtures with Alpine fahlore deposits like the Inn Valley (n = 2).

The final phase of the MBA (1200−1000 BC) is a transition period showing the end of the preceding South Alpine AATV dominance (4 of 12 objects) while foreshadowing the LBA with the arrival of leaded alloys with lead isotope ratios consistent with British lead ore and the revival of fahlore and an increase in the number of Sb-As-Ni-Ag bronze alloys. Three of the four remaining AATV items are presumably imports: a double-T handle knife from the NW German area (DB 1841 [125]), a Lüneburger-type spearhead (DB 911 [126]) and an Upper Danube *Vielwulstschwert* dredged from the Meuse River (DB 2450 [127]). The remaining eight objects are generally of mixed alloys and comprise mostly spearheads (n = 4), a razor (DB 1234 [128]), a socketed hammer (DB 2486), poorly dated casting debris (DB 3195) and an Atlantic sword from Zwijndrecht (BB 620 [129,130]).

### 3.3 Late Bronze Age/Early Iron Age (1000−600 BC)

#### 3.3.1 The LBA transition.

While there are clear lead isotope and source patterns in the MBA, the same cannot be said for the LBA. The main change between the late MBA and the LBA is the switch from Ni-As impurities (i.e., South Alpine AATV copper) to a broad Sb-As-Ni-Ag impurity profile. The source of LBA copper north of the Alps has long been a conundrum (e.g., [75,131]) and the research on the subject from Central European and Atlantic perspectives is highly fractured [83,132–135]. This – but also the underlying increased mixing and recycling – complicates the interpretation of the Dutch LBA objects.

**Fig 11:**
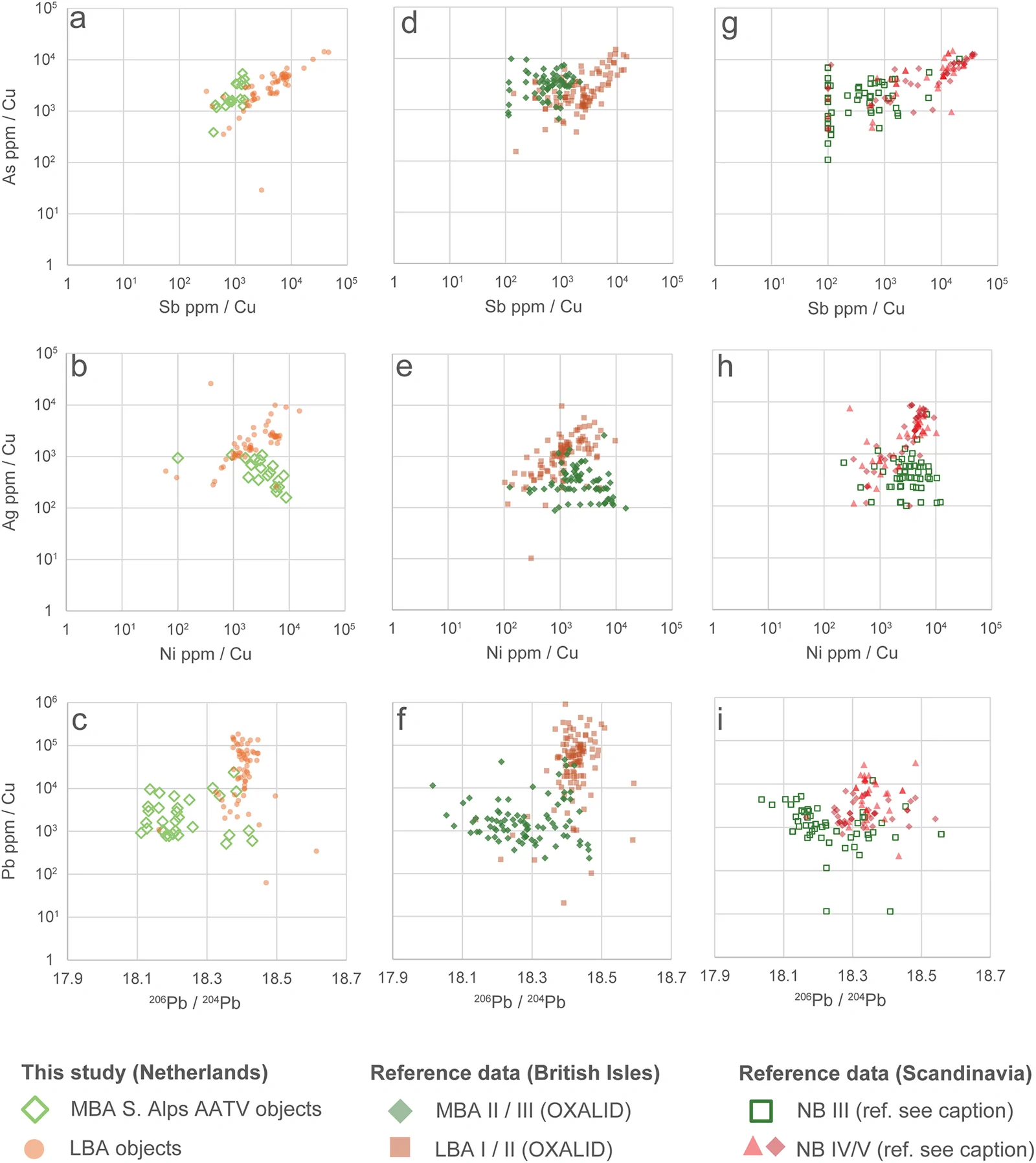
Elemental and lead isotopic changes occurring between the late Middle Bronze age and the Late Bronze Age by region. a-c) The Dutch late MBA is represented by the dominant South Alpine AATV group and all LBA objects are plotted. d-f) for comparison, MBA II/III and LBA I/II objects [55] represent the British Isles and g-i) contemporary data from Scandinavia from the Nordic Bronze Age III and IV/V [2, 3, 16, 136] (diagrams: S. W. Merkel).

The bronze used in the Netherlands in the Late Bronze Age is usually leaded, with lead contents above 2 wt. % (38 of 64), showing a commonality with contemporary British bronzes of the Ewart phase (ca. 1000−800 BC) [75], but the parallels with the British Isles go deeper. Both Dutch and British bronzes show very similar trace element (As, Sb, Ni and Ag) and lead isotope distributions in both the preceding late MBA (MBA II/III) and LBA (LBA I/II) ([55]; Fig 11; Fig H in S1 Appendix). This clearly shows the switch from Ni-As impurities (i.e., South Alpine AATV copper) to a broad Sb-As-Ni-Ag impurity profile. This switch is also visible in the Scandinavian bronzes occurring between Nordic Bronze Age (NBA) III/IV (1300−900 BC) and NBA V (900−700 BC) [2,3,16,136]. If these analysed Scandinavian objects can be seen as representative, leaded bronze occurs much less frequent there, and the concentrations of Sb, Ni and Ag appear to be even more pronounced in the NBA V than the British and Dutch LBA datasets (Fig 11).

What can be drawn from this is that the late MBA / LBA metal stocks used in Britain and the Netherlands are closely related while the LBA (NBA V) bronzes found in Scandinavia are more distantly related.

In the following discussion of LBA artefacts, the objects are divided by lead content into a lead-rich and lead-poor group using an arbitrary threshold of 2 wt. %. This threshold was chosen to be clearly beyond the previously proposed 1 wt. % threshold for copper [50] and beyond the lead content range of the MBA-B, which can occasionally be as high as 0.5-1 wt. %. Above 2 wt. %, the LBA objects exhibit a high degree of lead isotope homogeneity which is dissimilar to objects with less than 1 wt. % Pb. Based on this information, we can be fairly certain that for objects with lead contents above 2 wt. %, the lead isotope ratios reflect the influence of exogenic lead sources.

#### 3.3.2 Late Bronze Age: Lead-rich bronze.

There are two important features of lead-rich objects as a whole. Firstly, there is a trend towards a uniform minor/trace elemental pattern, generally following Sb>As>Ag ≈ Ni. All these elements are broadly correlated and are less than an order of magnitude apart in 32 of the 33 leaded bronzes analysed by ICP-MS (Fig I in S1 Appendix). The only leaded alloy that has a different elemental pattern (only As with low Ag-Ni-Sb) is the Nordic Type Oerel spectacle fibula (DB 2923 [46,120]), which also is distinctly unique because of its low tin content (1.6 wt. %). All other leaded bronzes contain between 4−16 wt. % tin and most between 8−14 wt. %. The elements Ag, Sb, As and Ni are negatively correlated with lead (r = −0.3 to −0.5), indicating that they are inherent to the copper metal and did not enter the alloy inadvertently with lead.

**Fig 12:**
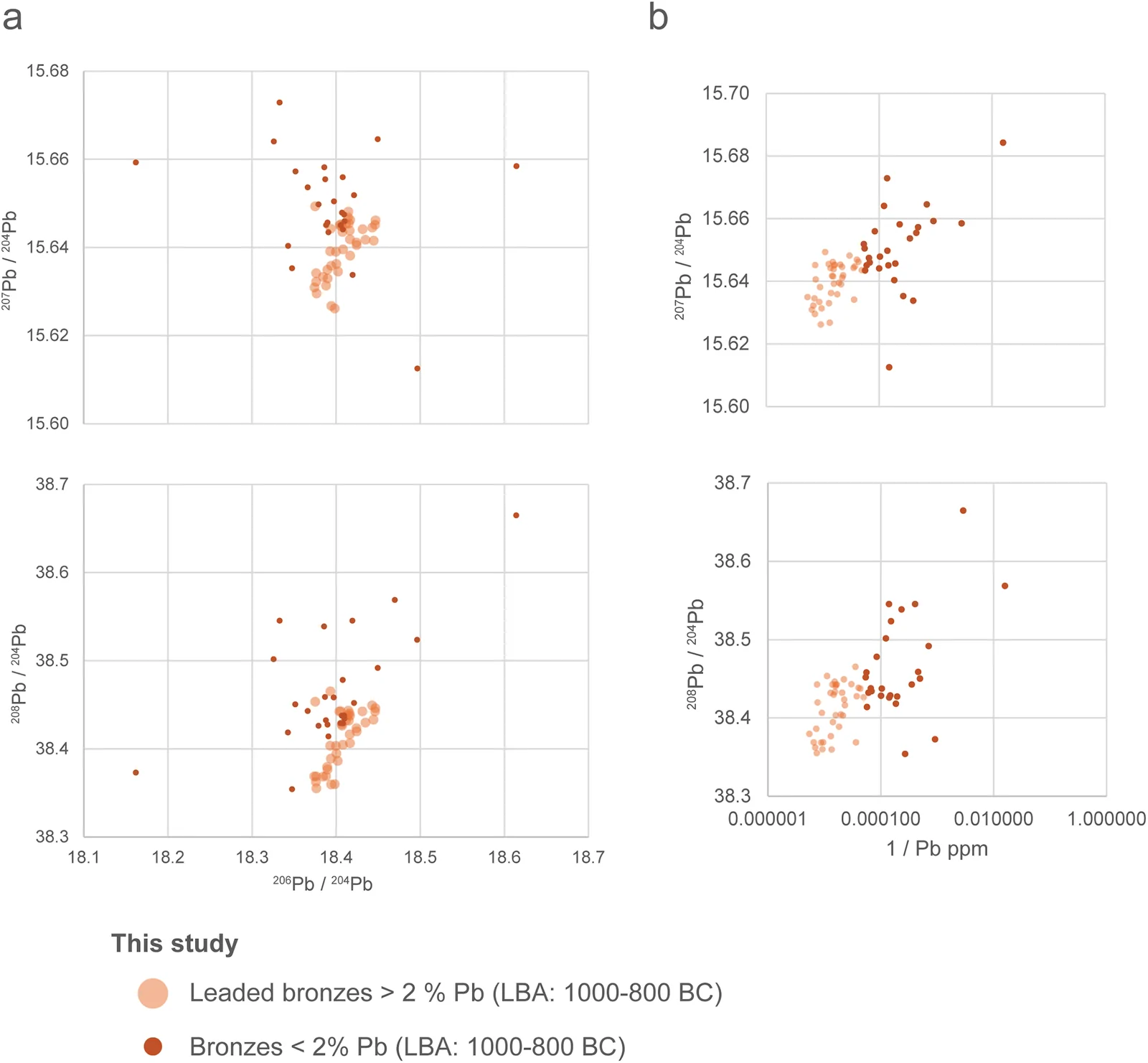
a) lead isotope ratios and b) lead concentration vs isotope ratios of LBA objects from the Netherlands divided by lead content (diagrams: S. W. Merkel).

The lead isotope ratios of these objects form a very narrow range in comparison to low-lead LBA bronzes and bronzes from earlier periods (Fig 12). The lead-rich objects, as a group, tend to plot lower in the isotope diagrams than the lead-poor LBA objects, thus pointing to a disjunction in source information. It is also evident in the leaded bronzes, but also to a degree in the bronzes with lower lead, that there is a relationship between lead isotope ratio and 1/Pb, particularly visible in the ^208^Pb/^204^Pb and ^207^Pb/^204^Pb ratios (Fig 12b) [137]. This is an indication of mixing between at least two lead sources within the leaded bronze group and possible mixing between leaded and low-lead bronze.

In the lead-rich group, socketed axes dominate (DB 2478 / DB 2745), with three originating from the LBA Oost-Maarland hoard (DB 3129 / DB 3131 / DB 3132 [44]). From the Markerwaardweg hoard [46], three items are present (DB 2921−1: a local omega-bracelet [138], DB 2925−2: a bronze plate necklace spacer and DB 2925: a Nordic Type Oerel spectacle fibula [120]). The remaining objects are a handle of an Atlantic Ewart-Park sword from the Vecht River (DB 928) and a stray chisel (DB 3187 [139].

**Fig 13:**
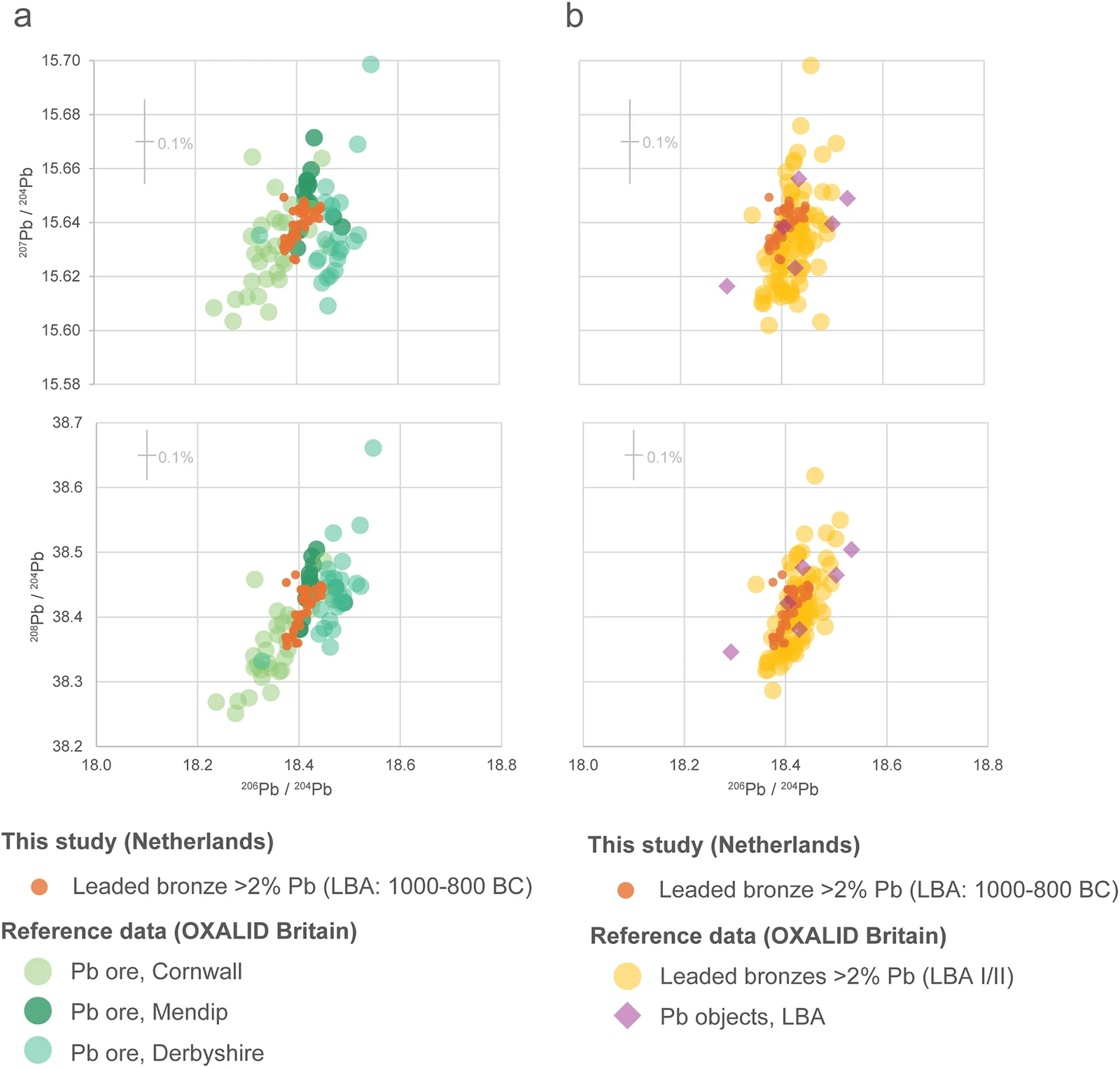
Lead isotope ratios of leaded bronzes from the Netherlands compared to a) lead ore from Cornwall, the Mendip and Derbyshire [55] and b) to LBA leaded bronzes and lead objects found in Britain [55] (diagrams: S. W. Merkel).

Because of the lack of correspondence between the minor/trace element patterns and the lead isotope ratios, together with the high lead levels not seen in earlier periods, it is safe to say that the source of lead in high-leaded bronze is not an indication of the copper source and cannot be expected to relate to known copper mines. Analyses of LBA leaded bronze artifacts and six LBA lead objects from Britain show a close similarity to leaded bronzes found in the Netherlands (Fig 13) [55]. Rohl and Needham [75] have suggested that Mendip lead may have been used in alloying in LBA Britain based on the lead isotope ratios of leaded bronze alloys particularly in the Wilburton phase (c.1150−1020 BC). There are speleothem data suggesting that the Mendip was mined for lead in the LBA [140] and Mendip lead ore do plot in the middle of the leaded alloy cluster, however, it is not sufficiently diverse to encompass the isotopic range of the leaded LBA alloys in Britain (e.g., Ewart phase c.1020−800 BC) or the Netherlands. Other British or continental lead mines must have also been active.

**Fig 14:**
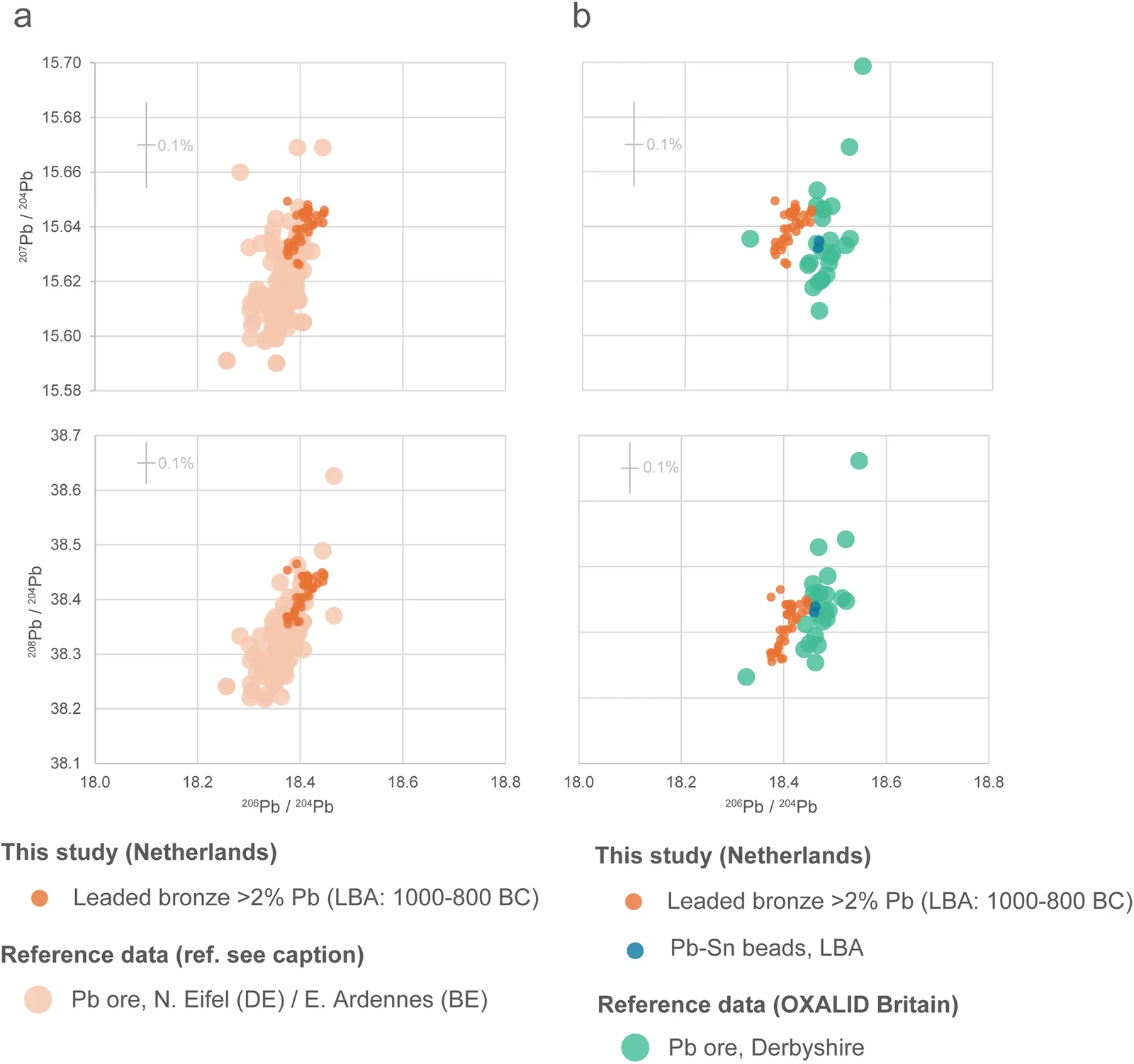
a) LBA leaded bronzes in the Netherlands compared with lead ore from the North Eifel [144–148] and the Belgian Mosan/Eastern Ardennes [149]; b) LBA leaded bronzes and two lead-tin beads in the Netherlands compared with lead ore from Derbyshire [55] (diagrams: S. W. Merkel).

The alluvial tin works of Cornwall and Devon are thought to be a major source of tin in the European Bronze Age [141] and Cornwall is also rich in primary lead vein deposits as well [75]. A mixture of Cornish and Mendip lead would align with the lower isotope range, while South Pennine/Derbyshire lead may be present in mixtures that would align with the higher isotope range (Fig 13) [55]. We suggest British lead sources because of an almost entire lack of LBA lead finds on the continental side of the North Sea, but, although admittedly scant, there are more lead artefacts known in Britain (e.g., [142,143] and recent Portable Antiquities finds). Despite the dearth of evidence, it is possible that geochemically similar post-Variscan deposits in German Rhenish Massif / Belgian Mosan / Eastern Ardennes [144–149] could have contributed to the lead supply to produce leaded bronzes (Fig 14a).

The only two lead objects analysed in our study, two lead-tin alloy beads from the Borger-Drouwenerstraat necklace (thought to be LBA-EIA: DB 3155–1/3 [48]) are consistent with Derbyshire lead ore, though none of the Dutch bronzes correspond to the centre of the Derbyshire lead isotope field (Fig 14b).

#### 3.3.3 Late Bronze Age: Lead-poor bronze.

The elemental compositions of the bronzes with less than 2 wt. % lead are more diverse. Five are Sb-rich fahlore (Sb/Cu > 1%) with varying Ni, two are Ag-rich alloys, two have As and low in other trace elements, one is Ni-As-(Sb) and the remaining 16 of 26 have all the elements, generally following Sb>As>Ag ≈ Ni (Fig J in S1 Appendix). Isotopically, the Sb-As-Ni-Ag bronzes show lower variability than the rest, overlapping with but centred slightly above the cluster of leaded bronzes in the lead isotope diagrams. These alloys do appear to represent elemental and isotopic mixtures. The one Ni-As-(Sb) bronze (DB 109, a basal-looped spearhead imported from the UK [17]) is consistent with the South Alpine AATV, while most of the Sb-fahlore and silver-rich copper alloys are broadly consistent with Slovakian and/or Inn Valley fahlore (Fig K in S1 Appendix). The two As only / low impurity copper/bronze objects are isotopic outliers (DB 3155, a conical bead and the Kirchloog socketed axe [150].

Ornament hoards strongly dominate the low-lead bronzes of this period, with nine objects originating from the Oost-Maarland hoard [44], four from the Nederviersel-Pulle hoard [45], three from the Markerwaardweg hoard [46] and three from the Eibergen hoard [47]. Amongst the other objects, two more ornaments are present (a Type Oerel spectacle fibula from Den Burg: DB 2848) and an everted palette bracelet from Dreumel [138,151]. It also comprises three Atlantic items: a Briester-Mâcon sword (DB 858 [152]); a type Nantes sword (DB 1721 [28]), both from the Meuse River, and a tentative UK socketed axe (DB 612 [153]). A spearhead with ribbed socket from Erica may present a local product or import from adjacent Germany (DB 128 [154]). Other presumable local items may be a bronze mould for casting Type Helmeroth socketed axes (DB 643: from the Meuse near Roermond [155]) and a socketed axe of Type Wesseling from Molenaarsgraaf, radiocarbon dated by its ash haft (DB 3056: c. 901−796 BC [156]).

#### 3.3.4 Early Iron Age.

Five objects belong to the final phase of our study. The alloys are extremely heterogeneous, mostly consisting of Sb-rich fahlore and leaded alloys, although the lead isotope ratio range fully fits within the LBA Sb-As-Ni-Ag bronzes and leaded bronzes. Good parallels are the LBA/EIA hoards of Jodłowno, Pomerania, and Mariesminde, Denmark, which consist mostly of leaded and fahlore-type alloys and partially share the same lead isotope range [157,158], but the Dutch objects’ lead isotope ratios have a greater affinity to earlier Dutch LBA leaded bronzes and thus a stronger connection the British and/or Rhenish lead sources.

Two Type Gündlingen swords are parts of this group, which are crafted from fahlore (DB 1420 [159]) and diluted fahlore respectively (DB 1618 [160]). Moreover, it comprises a diluted fahlore sickle from a hoard of flint sickles at Heiloo (DB 508 [49]) and a poorly datable decorated neck ring from a bog near Fochteloo (DB 206; with 18 wt. % Pb). The final object is an Italian type ‘Leech’ fibula, whose Dutch provenance is questionable (DB 233 [120]).

#### 3.3.5 Source(s) of LBA bronze: Atlantic vs Central Europe.

The data produced in our study contribute to the current debate on the role of Iberian metal sources and the maritime Atlantic trade routes on the bronze supply of Northern Europe [5].

The low variation in the Sb-As-Ni-Ag element profile of nearly all lead-rich bronzes and most low lead bronzes suggests high-intensity mixing of bronzes produced from fahlore-chalcopyrite mixtures potentially together with low impurity copper. This conclusion has already been drawn for the British LBA [132]; and our data indicate this is similarly the case for the Netherlands.

In the LBA, it is generally accepted that Sb-As-Ni-Ag copper has its roots in the Alps [161]. It has been shown that large-scale blending of chalcopyrite and fahlore copper already had been occurring in the north of the Alps in the LBA [95,134,162,163]. Examples of Alpine chalcopyrite sources could be Kitzbühel, Mitterberg [95] and the South Alps AATV [103] and fahlore could be from the Inn Valley [95].

**Fig 15:**
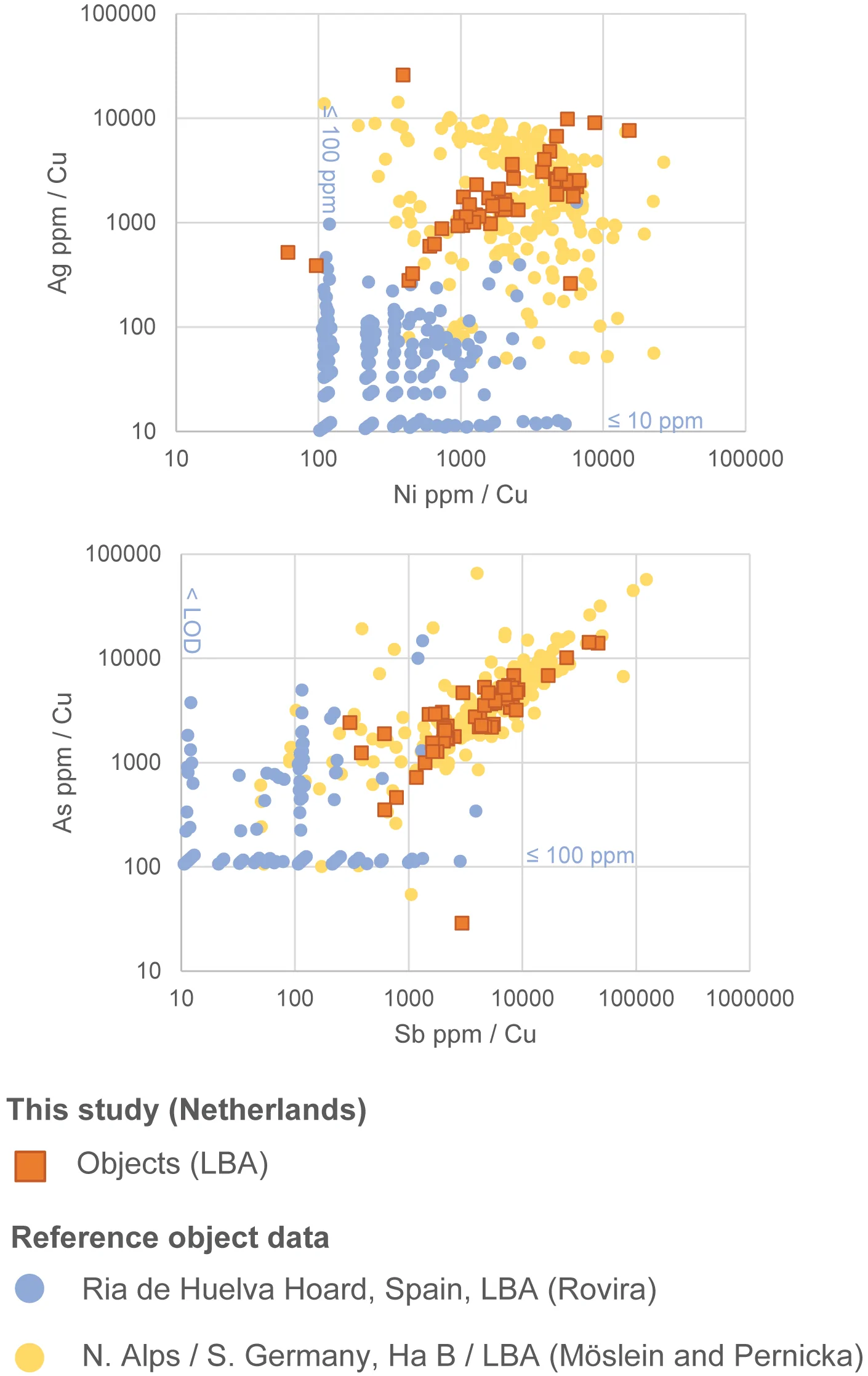
Trace element patterns of LBA bronzes found in the Netherlands compared to LBA bronzes from the Ría de Huelva metal assemblage from Spain (n = 326; [165]) and bronzes from the Northern Alps and Southern Germany from the Hallstatt B period (n = 206; [134]) (diagrams: S. W. Merkel).

**Fig 16:**
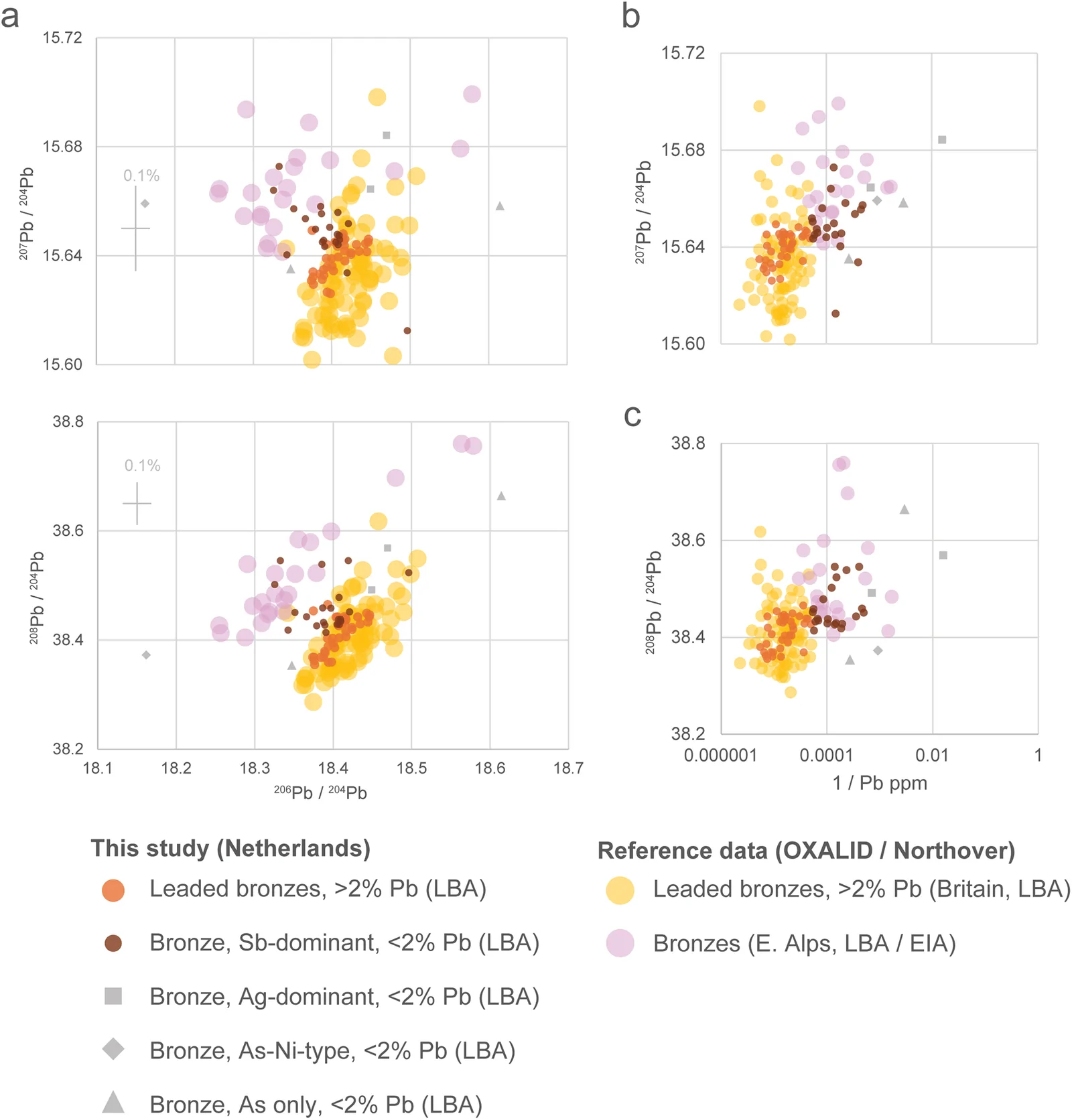
a) Lead isotope ratios of LBA bronzes found in the Netherlands compared with leaded bronzes in Britain [55] and mixed low-lead bronzes from the Austrian Eastern Alps [162], and b-c) lead concentration vs lead isotope ratios of the same bronzes (diagrams: S. W. Merkel).

In stark contrast to the high impurity Sb-As-Ni-Ag copper from the Alps, Iberian LBA bronzes have low levels of trace elements [161]. One of the largest LBA hoard assemblages in western Europe, the Ría de Huelva hoard, dated to the second half of the 10^th^ century BC [164], was analysed in the 1990s [165]. The low impurity trace element profile of the Huelva hoard is confirmed by more than 300 object analyses and is inconsistent with LBA bronzes from the Low Countries (Fig 15). Instead, the trace element pattern seen in LBA bronzes in the Netherlands are directly comparable to the vast majority of analyses of Hallstatt B bronzes from Austria, Switzerland and Southern Germany (Fig 15) [134,162]. The lead isotope distribution for the bulk of Dutch LBA objects could represent the mixing between lead from British/Rhenish lead deposits and Alpine bronze, also characterized by a Sb-As-Ni-Ag elemental profile (Fig 16) [162].

The same relationships in Dutch-found LBA bronze and Alpine metal cannot be found in Iberian LBA metalwork [164–171] and Sardinian metal objects [172,173]. Since the chemical analyses of existing LBA Iberian artefacts are markedly different (low impurity) from the LBA Dutch artefacts and those from the surrounding regions (high impurity Sb-Ni-As-Ag), it is misleading to compare these high-impurity objects to lead isotope data of Iberian copper ores even if there are isotopic overlaps.

The greater bulk of LBA copper used in the Netherlands and in Britain is more closely related to Central European/Alpine metal stocks than Iberian Atlantic and Mediterranean. Yet indisputable LBA evidence for contact along the Atlantic coast exists between Iberia and Northern Europe, such as sword typology and rock art [133,174]. Double looped palstaves, which are usually leaded bronze [175], are found along the Atlantic and North Sea coast as far as the Netherlands, indicating direct Atlantic contacts in the LBA [176]. Analyses of this type of leaded palstave in NW Iberia indicate that lead from southern Spain (Sierra de Gádor) was used in their manufacture [161], but, without exception, the lead used in all Iberian leaded bronzes is different from the lead in leaded bronzes in the Netherlands (Fig L in S1 Appendix) [55,166–171,177–179]. Thus, despite contact, Iberia and the North Sea largely belonged to different spheres of metal circulation.

While Atlantic contacts existed, this is not necessarily directly visible in the wider raw material pool for LBA metalwork in the Netherlands. However, Iberian copper/bronze may have entered the metal supply through mixing with high-impurity bronze or rarely as low impurity objects. The LBA “arsenic only” Plainseau-style socketed axe, which were deposited by the thousands in Southern Britain and Normandy [150], has lead isotope ratios consistent with Ría de Huelva bronze [55,168,180] but not exclusively. The lead isotope ratios of this object are also consistent with the Great Orme and deposits in Cornwall [30,55,79] and thus could reflect British LBA copper production. This highlights the difficulties in demonstrating indisputable metal supply links between Northern Europe and the Atlantic seaboard in the LBA.

## 4 Discussion

**Fig 17:**
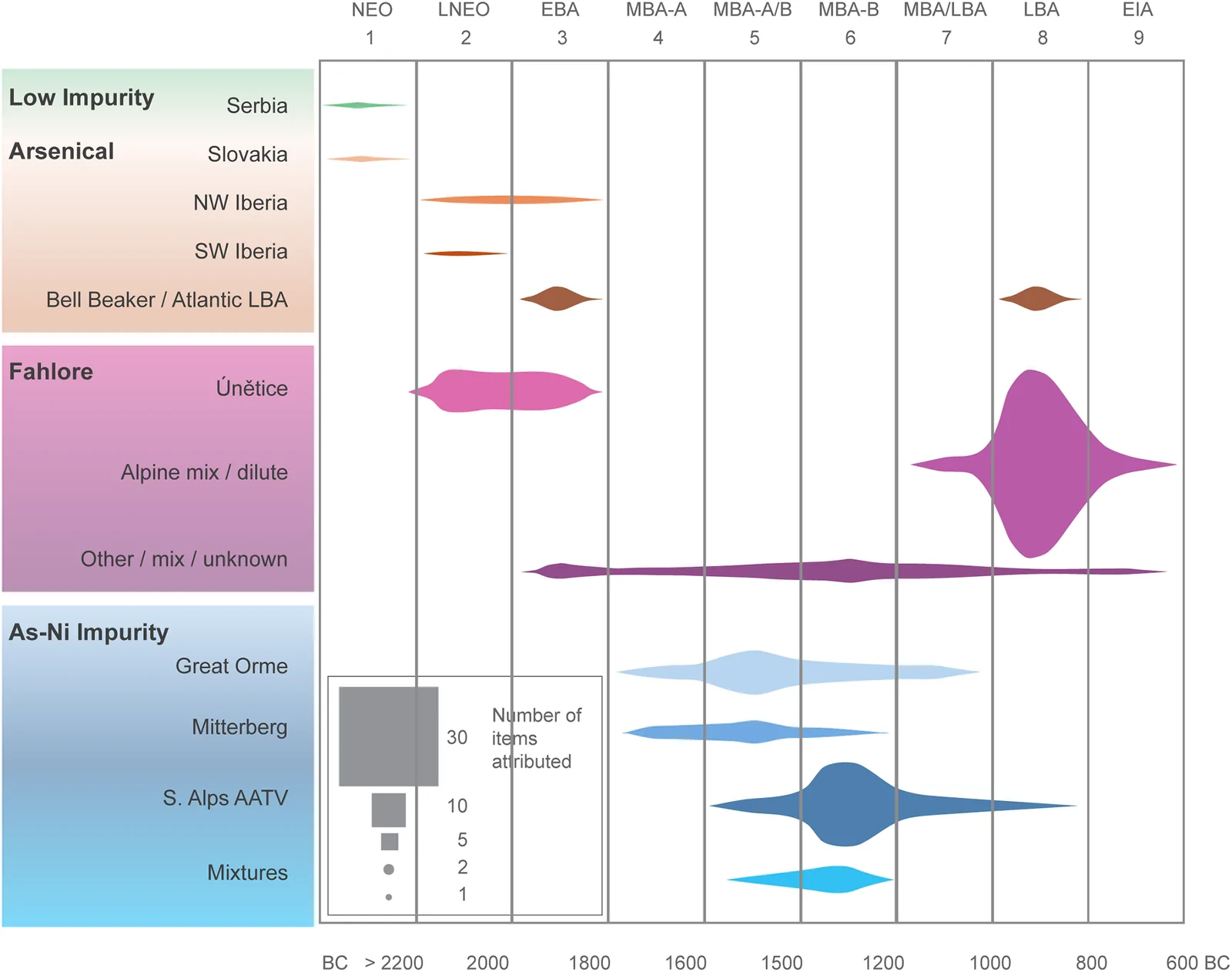
Chart showing the chronological distribution of metal objects divided by metal stock/source group (diagram: S. Arnoldussen, S. W. Merkel).

Based on the above analyses, we can plot the diachronic shifts in number and dominance of metal flows into the Netherlands (Fig 17).

**Fig 18:**
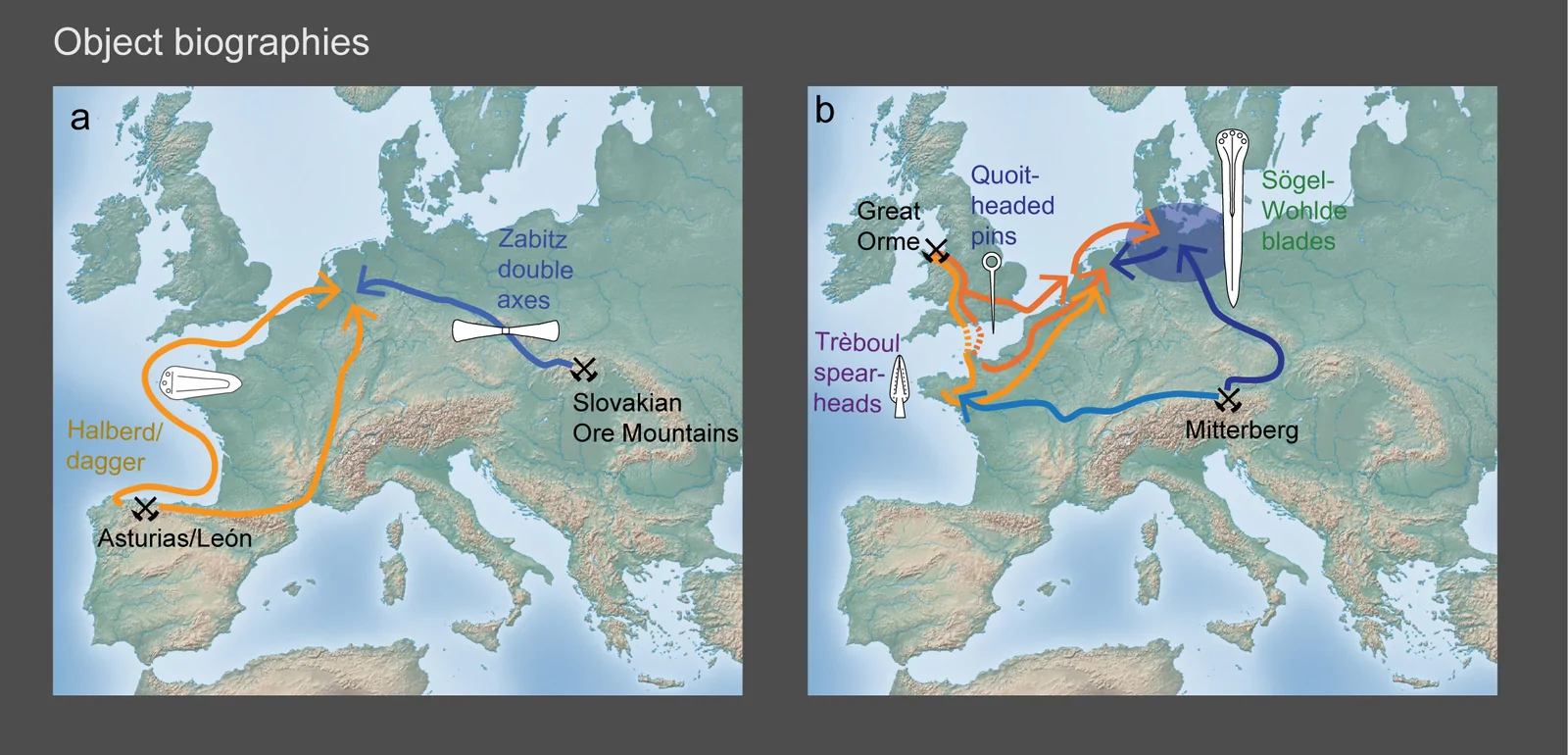
Maps showing some possible routes for metal object transport in the Late Neolithic (A) and in the Middle Bronze Age A (B) (maps: S. Arnoldussen, S. W. Merkel). Base map © Esri. Sources: Esri, TomTom, Garmin, USGS, FAO, NOAA. Map image is the intellectual property of Esri and is used herein under license. Copyright © 2026 Esri and its licensors. All rights reserved. Reprinted under CC BY license with permission from ESRI (https://doc.arcgis.com/en/arcgis-online/reference/static-maps.htm).

The earliest copper (alloy) artefacts from the Low Countries are most likely imports from central Germany. The double axe of type Zabitz-Westeregeln (DB 613), made from Slovakian arsenical copper, has its region of origin in central Germany and is the sole example of this type in the Netherlands [61] (Fig 18a). The Glanerbrug pure copper flat axe (DB 1352) was possibly made from Serbian ore, and – due to the absence of a sharpened cutting edge – may be representative of the way in which unalloyed copper moved across Europe, as axiform ingots or roughouts. There is no evidence for local production in the Netherlands at this phase. With the period of the Bell Beaker culture groups, copper items such as awls and daggers are commonly part of the funerary assemblages [18]. These were generally crafted from both high impurity fahlore copper with parallels to the Únětice Culture and arsenical alloys with low levels of Sb, Ag and Ni interpreted as being connected to the EBA Bell Beaker culture spread over the European Atlantic [6,71].

The Wageningen hoard – tentatively dated here to 2200−2000 BC – is a salient marker of the next phase of copper alloy networks in the Netherlands, as it combines both arsenic coppers and tin bronzes. The majority of the items in the Wageningen hoard are crafted from Únětice/Slovakian-type alloys, but the big triangular blades (one halberd and one dagger) were crafted from Iberian arsenical bronzes (Fig 18a). Notably, the only other halberd sampled (DB 1792, from Roermond), was also crafted from Iberian arsenical bronze. The fact that the Wageningen halberd was affixed with rivets from Slovakian fahlore copper, suggests the blade was imported separately.

For the first two centuries of the 2nd millennium BC, the number of objects directly identifiable as evident imports (in the present as well as in the past) remains striking. This suggests that intact object imports were still an important source of metal influx in the Early Bronze Age. For example, the Drachten (DB 2862) and Haren (DB 1697) flat axes were both decorated in styles common to the British Isles (and both possibly crafted from Iberian metal). Other examples are the Armorican dagger (DB 2856, from Iberian metal) from Donderen or the Swiss/S.German *Prunkbeil* from Hilversum (DB 2095, from Únětice/Slovakian metal). In addition to such clear imports, a (supra)regional tradition of low-flanged axes emerged in Western Europe around this time, some of which may have been (re)cast locally (as indicated by recognisability of local types and rare indications for mixing of alloys). These axes, often classified as Type Emmen and related variants [61] are crafted from Bell Beaker/Iberian (n = 5) as well as Únětice/Slovakian metal stocks (n = 15), testifying to the two main influxes of metal for the period of 2000−1800 BC.

With the start of the Middle Bronze Age, and particularly the 16th century BC, two novel cultural affinities strongly come to the fore: a (Nordic) Sögel-Wohlde weapons complex and an (Atlantic) maritory network in which – amongst others – Normandy Type Tréboul spearheads were exchanged (Fig 18b). The four swords and two daggers attributed to the Sögel-Wohlde style groups reflect a new availability of metal: three were crafted from Austrian Mitterberg-type copper, two from Welsh Great Orme-type copper and one from Italian South Alps AATV metal. The Tréboul spearheads, similarly, reflected the availability of Great Orme (n = 3) and Mitterberg (n = 2) metal. This indicates that Mitterberg and Great Orme copper alloys were reaching both the Nordic and Atlantic realms, where they were cast into objects of decidedly regional styles. It also shows that the previously important Slovakian fahlores played just a minor role and this shift hints at a restructuring of the underlying exchange networks.

In the second half of the Middle Bronze Age, the Italian South Alps (AATV) copper would become dominant, albeit with few objects consistent with Great Orme and Mitterberg metal. For this phase, an increasing number of objects show evidence of mixing, in some still recognisable combinations of Italian AATV, Mitterberg and Great Orme-type copper – and with some objects showing fahlore of unknown origin being added. This intensified mixing, combined with the evidence of moulds, suggest an intensified local production that in turn probably relied on an increased demand for raw materials. The shapes in which these raw materials entered our areas were both (scrap) palstaves from the Atlantic network [94,121,181] and as rough-cast palstave-shaped axiform ingots [101]. Palstaves – be it imported or locally (re)cast, were mainly (n = 12) cast from AATV metal, or combinations of AATV and Mitterberg-type metal (n = 4). Only a single specimen (DB 2772) was crafted from a fahlore originating from either Slovakia or the Inn valley area.

The Ommerschans hoard, featuring the spectacular aggrandized dirk of Plougrescant-Ommerschans type (DB 1758 [115]) also reflects the dominance of AATV-type copper, as all but two items (a chisel and pin; DB 1761/1763) were cast from AATV metal. The Jutphaas blade (DB 1823) of Plougrescant-Ommerschans type was also made from copper originating in the AATV. This means that the wider group of Plougrescant-Ommerschans blades contains objects perhaps crafted from Great Orme metal exclusively or as mixtures with AATV metal (Kimberly and Oxborough dirks [182]) and examples crafted from metal consistent with the AATV (Beaune [182]) and those from our study), thus showing heterogeneity of sources in a stylistically tightly related group of blades. This suggests that moulds or objects travelled and copies were made wherever there was a demand.

While the start of the Late Bronze Age saw many innovations in novel types and forms of objects, the Alpine sources remain dominant, albeit almost always as mixtures of chalcopyrite and fahlore. Isolated exceptions are an Atlantic sword from Zwijndrecht (BB 620, possibly made from Great Orme metal) and a *Rahmengriff* razor (DB 1234) from fahlore potentially from the Inn valley. Inn valley metal appear to have been used in the Late Bronze Age (1000−800 BCE). Leaded alloys – despite occasionally being used in the preceding period (e.g., DB3156; DB 3188) – are now commonplace, with isotope ratios suggesting British lead usage for the LBA alloys. The two lead beads of the necklace of Borger-Drouwenerstraat (DB 3155–1/3) appear to be the earliest use of lead in the Low Countries, which was likely a rare and ‘exotic’ material. The lead ring on the Mindelheim sword of Oss, iconic for the start of the Early Iron Age in the Netherlands, is the only other presently known example of the use of lead [183]. Also in Britain, prehistoric objects made from lead have been found but are rare. Examples include a recent LBA lead ingot from Shropshire wrapped around two gold lock rings [184] and three rectangular lead ingots that were recently found at Dereham in Norfolk in a hoard context together with four socketed axes, and other objects date to the Late Bronze Age 950−800 BC [185]. Another Bronze Age lead ingot was excavated at Runnymede Bridge [186], a lead palstave was found near Maidstone in Kent [142] and a lead necklace in Scotland [143]. A small lead alloy cone was found in a burial pit as part of the funerary deposits made in the 9th century BC at Cliffs End Farm, Isle of Thanet, in Kent [187]. Excavations on the hillfort of Mam Tor in 1969 yielded a fragmented axehead, probably from a socketed axe, made from lead [188]. Evidently, whereas prehistoric objects of pure lead form rare occurrences to either side of the channel, the amount of what we argue to be British lead in Dutch bronze alloys suggests intensive contacts during the Late Bronze Age. Moreover, it raises the intriguing question of where the workshops were located where such lead was added.

Large hoard assemblages such as the Nederviersel-Pulle hoard [45] from which 14 objects were studied, contains objects only made with dilute fahlore but with varying lead concentrations, showing that swords, spearheads and axes could all be cast from such alloys and that the degree of mixing was severe.

## 5 Conclusions

**Fig 19:**
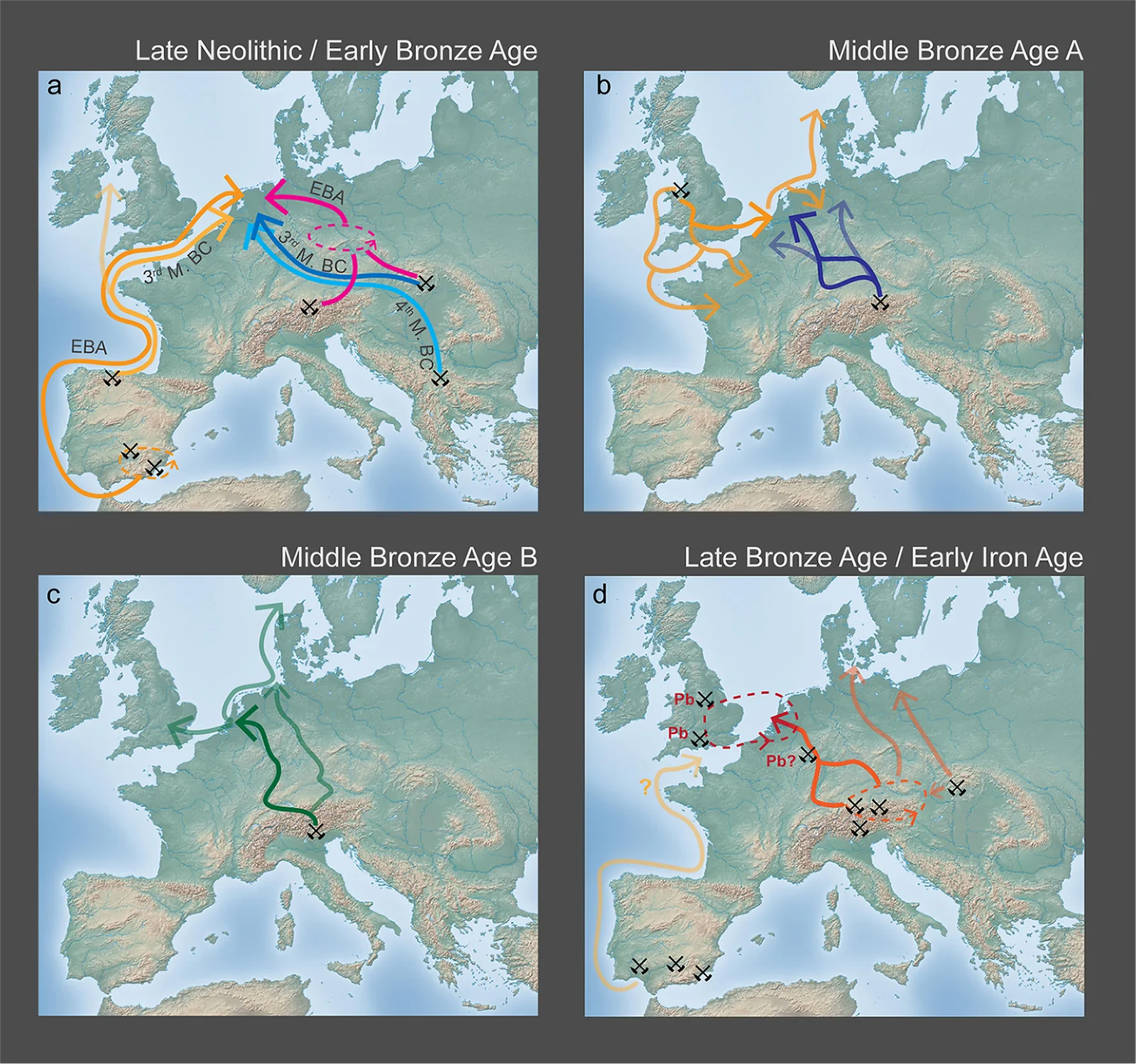
Reconstruction of metalwork flows for the different phases (a-d) and specific objects (e-f). The routes suggested include the use of the major river systems (Rhine, Weser, Elbe, Oder and their tributaries) (maps: S. W. Merkel). Base map © Esri. Sources: Esri, TomTom, Garmin, USGS, FAO, NOAA. Map image is the intellectual property of Esri and is used herein under license. Copyright © 2026 Esri and its licensors. All rights reserved. Reprinted under CC BY license with permission from ESRI (https://doc.arcgis.com/en/arcgis-online/reference/static-maps.htm).

By embedding the new data from the Netherlands into the corpus of existing data, we are able to significantly expand and reshape interpretations on Bronze Age metal movement. Our study reveals non-linear and dynamic evolution of metal flow patterns (Fig 19).

Particularly in the earliest periods (Figs 18a and 19a), arguments for the long-distance movement of individual metal objects be made, for example late Neolithic axes and arsenical bronze halberds, but even by the EBA, there appear to be metal circulation pools in both the Únětice cultural area of Central Europe and the Atlantic Bell Beaker culture. The Netherlands, being on the periphery of both cultural groups, tapped into both metal stocks. This is embodied by the Wageningen hoard, which not only is a collection of metal from these two different cultural origins, but its composite halberd (Iberian metal source) and rivets (Únětice metal source) reflect this cultural “betweenness” in a single object.

While in the EBA there are objects that are clearly made from fresh metal and probably direct long-distance imports, the general Dutch metal supply appears to have a “second-generation” character, possibly homogenized through casting and circulation in the core cultural areas before making their way to the Netherlands. In other words, an indirect diffusion of metal through culturally related areas. Though the process of homogenization in the core cultural areas removes extreme compositions and breaks direct links to mines supplying the metal, the process gives the metal a new culture-based identity, which is valuable in reconstructing metal flow.

In the early MBA (Figs 18b and 19b), the Netherlands again finds itself between two inflows of metal, one from the Great Orme in Wales and the other being the Mitterberg in the Austrian Alps. It is not possible to know how long the metal was in circulation before it arrived in the Netherlands, but it is clear from the sharp elemental and isotopic patterns that there was little mixing of sources and extraneous metal rarely entered the metal supply. As the growing number of analyses indicate, continental products like Tréboul-type spearheads, Nordic Sögel-Wohlde blades and low flanged axes of Mägerkingen type can be made of either Great Orme or Mitterberg copper, which means that societies in Northern France, the Netherlands and Northern Germany and Southern Scandinavia received metal from both sources. The elemental and lead isotope evidence suggests that the MBA-A is a period of stable metal flow from mining regions to distant metal workshops.

This system broke down by the MBA-B (Fig 19c), when there was an increase in the number of obviously mixed-source alloys and a gradual displacement of older copper stocks by metal coming from Italian South Alpine AATV sources. The South Alps AATV eventually becomes the main source of metal for the European Continent to the north of the Alps achieving almost monopoly conditions during the later Middle Bronze Age. It is the dominant source of new metal in the MBA-B in the Netherlands, a prominent example being the Ommerschans hoard, which almost exclusively contains metal consistent with the South Alps AATV. The Netherlands may have played an important role in the transport of South Alps AATV metal via the Rhine for oversea export to the British Isles and possibly also Scandinavia.

Our data indicate that the Late Bronze Age (Fig 19d) is a period of high intensity circulation and mixing of bronze in Europe north of the Alps. The high impurity Sb>As>Ag ≈ Ni bronze compositions which become dominant in the Netherlands and Britain have their origins in the Alps, where LBA Alpine bronzes – already mixtures copper from diverse Alpine chalcopyrite and fahlore sources – share the same trace element and lead isotope patterns. This is in strong contrast to Iberian bronzes which are low impurity copper. LBA Iberian copper sources were not major suppliers to the Low Countries, which firmly discredits the theory of a direct sea route supplying Scandinavia and the Baltic with Iberian metal and highlights the need for re-evaluation of Scandinavian and Baltic datasets considering Central European fahlore sources and mixtures. Beyond copper, leaded bronzes become commonplace and the lead isotope evidence suggests a shared pool of metal between the Netherlands and Britain using British and/or Rhenish lead sources for lead alloying.

The general trends are in many respects similar to the results of chemistry and lead isotope studies based on Scandinavian material [83], especially Danish [3] and Swedish datasets [2], but there are also differences. During Late Neolithic and Early Bronze Age societies in Denmark and the Netherlands were supplied with copper from the Slovakian Ore Mountains, as a major source. Additionally, Dutch Bronze Age communities also acquired items made of Iberian arsenical copper/bronze, whilst Denmark obtained objects made of copper from Welsh and English mines [4]. In the Middle Bronze Age, copper from the Great Orme mine became important, but there seems to be a difference in intensity and scale as it was it was more intensively used for objects in Sweden and the Netherlands than in Denmark [83]. The major shift in trading routes, around 1500 BC, towards the Mitterberg mining area and the Italian Alps, is also a common trend, for the societies in Scandinavia as well as in the Low Countries. Comparative studies of this kind, focusing on the intensity and connectivity of the various exchange networks, are promising projects for the future.

The narrative synthesis of the Bronze Age societies in the Low Countries starts with a formative phase, up to 2200 BC. At this stage, copper and bronze objects were rare, strange and exotic. The Bronze Age people acquired them as exchange items as gifts and subsequently – upon reception – didn’t remelted them, but rather left the items intact, deliberately in the landscape, mostly as single depositions. In the period 2200−1800 BC, copper/bronze flat axes became the norm. These axes were highly sought after and intensively exchanged at North-Western European scale. For everyday people, bronze became a more common metal, present in kinship groups and used in dwelling places. Bronze was valued as useful material and its practical properties rise to the fore. During 1800−1600 BC interactions in exchange networks seems to have intensified, with blades and imported spearheads as new items, besides an increasing number of axes. Participants in this long-distance exchange include groups in Brittany and Wales, as well as in Northern Germany. More people were connected, but distantly, via communication by water, exploring both coastal and riverine routes, and over land. They never met in daily life and the objects travelled through areas that were never visited by the receivers. In the period 1600−1200 BC, evidence for local metal working (moulds, crucibles) is emerging but is still limited. The craft of casting bronze is becoming increasingly known within the Bronze Age communities, as is having or at least knowing a bronze smithy within the kinship groups. Bronzes are very common, ever expanding the repertoire of forms (swords, tools, sickles, pins, razors, etc.). People are also exchanging items that were made from mixed or recycled bronze, including more regional types, implying shorter barter distances between the groups. In the period of 1200−800 BC, the variety of metalwork expanded dramatically, both in terms of numbers and new designs. New fine decorated objects, such as bracelets, and lost-wax casting (*cire perdue*) require new alloys, and leaded bronzes were introduced. Melting, mixing and recycling of items became common practice. People were more strongly connected in exchange networks, with groups in Brittany (Atlantic) and Denmark (Nordic), and interactions intensified. The obtained objects were very commonly discarded or intentionally removed from circulation, especially in axe-tokens hoards [189].

Despite the absence of rich bronze cultures in the seemingly empty Low Countries, the material is crucial in understanding metal flow networks of this period. The Netherlands as a North Sea maritime and major delta region was strongly connected and was a conduit for far-reaching exchange networks. This Dutch connectivity has its own distinct character; there are no direct links to Ireland or Sardinia, contrary to previous assumptions based on typological grounds, reminding us to be careful to not confuse typological/stylistic labelling with metal source.

The results reveal the relationships between the Dutch artefacts (and their final context of deposition, grave or settlement) and the specific mining regions and metal stocks. The different origins of copper, tin and lead, the ‘intermediate workshop locations’, the complexity of the interactions and the means of transport behind the routes are much less clearly defined. These gaps in knowledge raise new thought-provoking questions: where were the workshops located, to what extent were bronze objects locally produced in the Netherlands? And how did the lead metal flow between England and the Low Countries? This also calls for a different type of research and approach. In practical terms, the Dutch archaeological community should be more alert to contexts where there is a high probability of finding boats (coastal dunes, wetlands) and subtle traces of metalworking (settlements and settlement peripheries). In addition to this local awareness, future joint studies on a European synthetic level will enrich our understanding of the Bronze Age.

Overall, this study highlights the value of combining and critically evaluating new and legacy datasets to explore data trends across borders. We hope that the research presented here forms a building block for future studies on the development and evolution of metal flows across the European Bronze Age.

### Sample information

No permits were required for the described study, which complied with all relevant regulations.

## Supporting information

S1 Appendix Supplementary figures. (PDF)

S2 Appendix Standards and data quality. (PDF)

S3 Table ICPMS standards. (XLSX)

S4 Table Pb isotope standards. (XLSX)

S5 Table Sample data. (XLSX)
