# Supplementary material for: Tracing Europe-Wide Copper and Bronze flow in the Dutch Late Neolithic to the Early Iron Age
Source: PLoS One. 2026 Sep 8;21(9):e0348751. doi: 10.1371/journal.pone.0348751 (PMC13552965; doi:10.1371/journal.pone.0348751)
Supplement: Supplementary file 1 [file pone.0348751.s001.pdf]

Tracking Europe-Wide Copper and Bronze Flow in the Dutch Late Neolithic to the Early Iron Age

Stephen Merkel, Stijn Arnoldussen, Liesbeth Theunissen, Bertil van Os, Judith van der Leije, Joris Brattinga, Luc Amkreutz, Bibi Beekman, Irini Biezeveld & R. Alan Williams

S1 Appendix. Supplementary figures

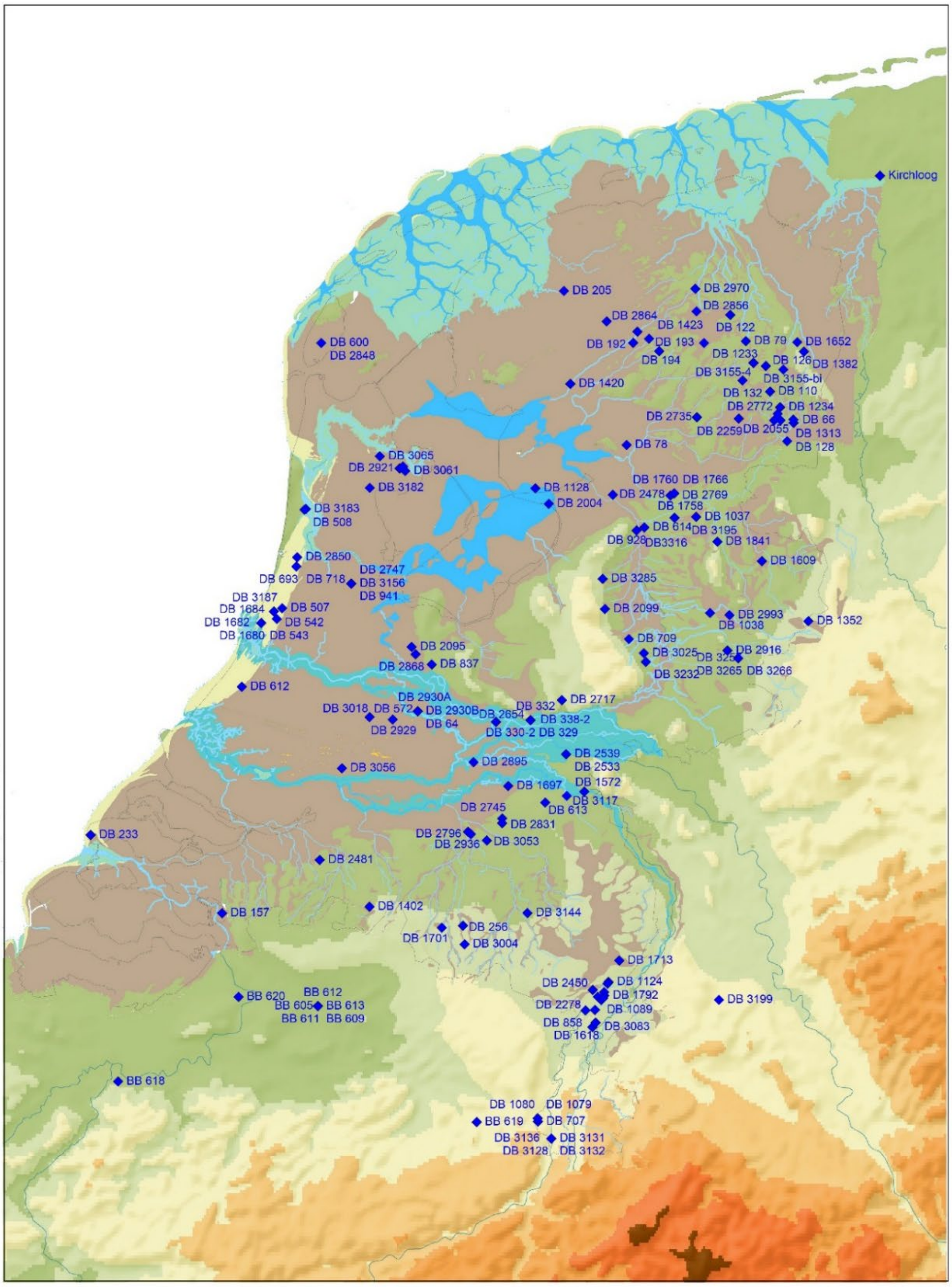

Fig A. Find location of the objects investigated in this study overlayed on a map of the Netherlands with the landscape terrain from around 1500 BC (map: S. Arnoldussen, S. W. Merkel; Base map: Vos, P., M. van der Meulen, H. Weerts and J. Bazelmans 2020: Atlas of the Holocene Netherlands, landscape and habitation since the last ice age, Amsterdam. Reprinted under CC BY license with permission from the Rijksdienst voor het Cultureel Erfgoed, original copyright 2019). (Link: https://www.cultureelerfgoed.nl/documenten/2019/01/01/paleogeografische-kaarten-zip).

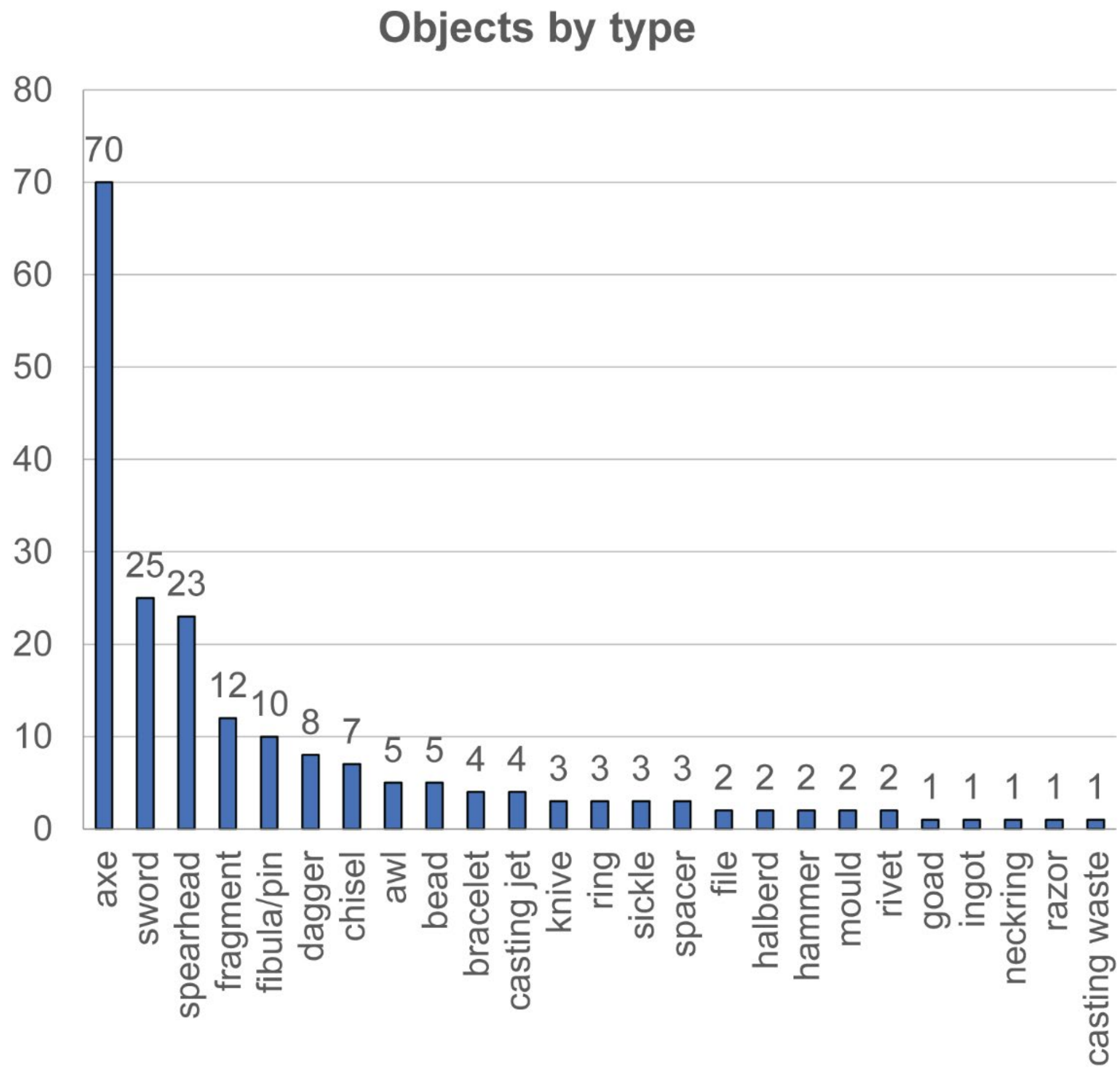

Fig B. Objects by object type (diagram: S. Arnoldussen, S. W. Merkel).

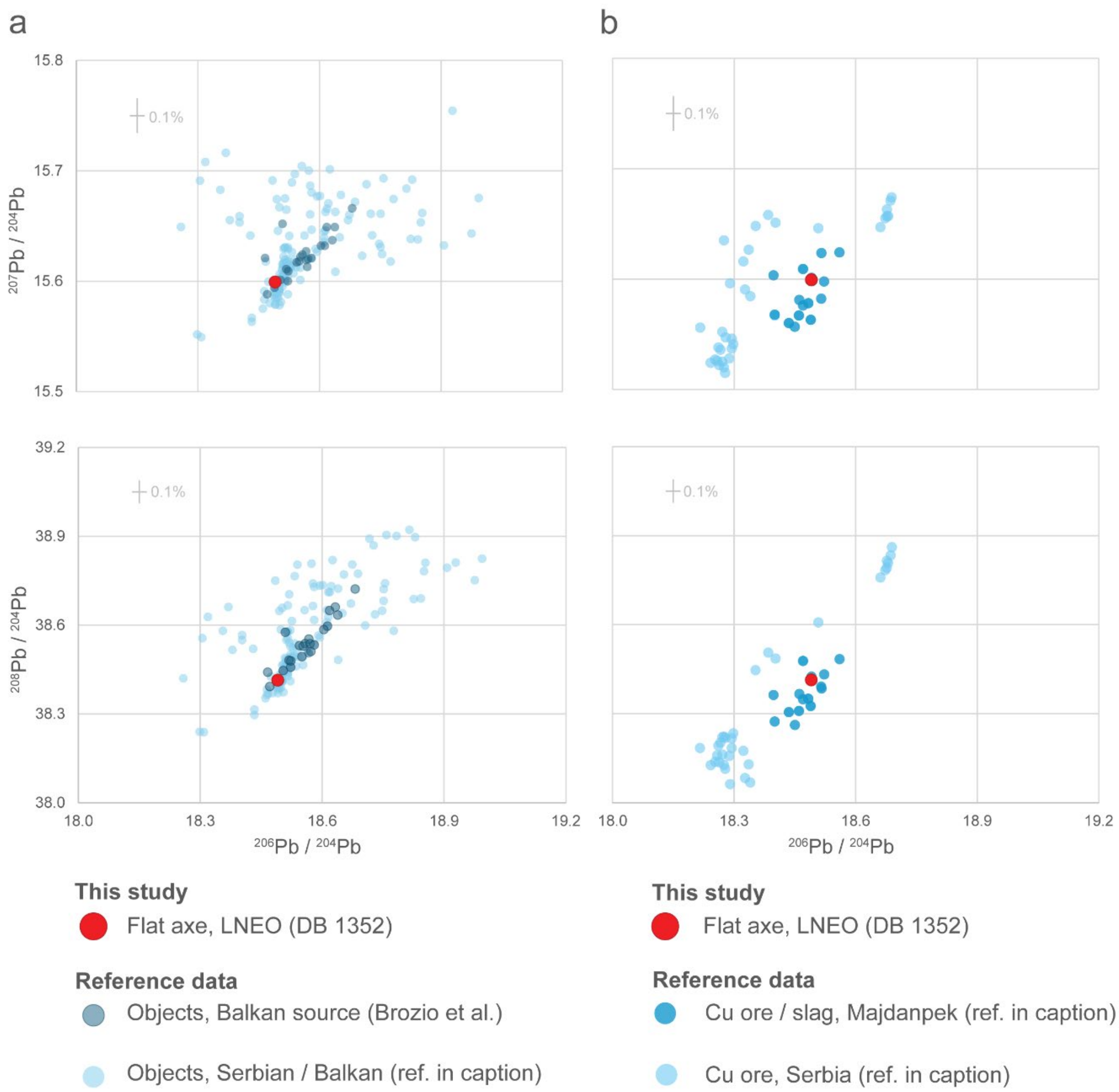

Fig C. a) lead isotope ratios of a Late Neolithic copper flat axe (DB 1352) compared to contemporary copper objects from Serbia and the Western Balkans [62,64] and objects attributed to Serbian copper sources [65]. b) the same axe compared to copper ore and slag from Majdanpek [62,63] and other copper deposits in Serbia [62,66]. Lead isotope ratio errors are expected to be less than 0.1% (diagram: S. W. Merkel).

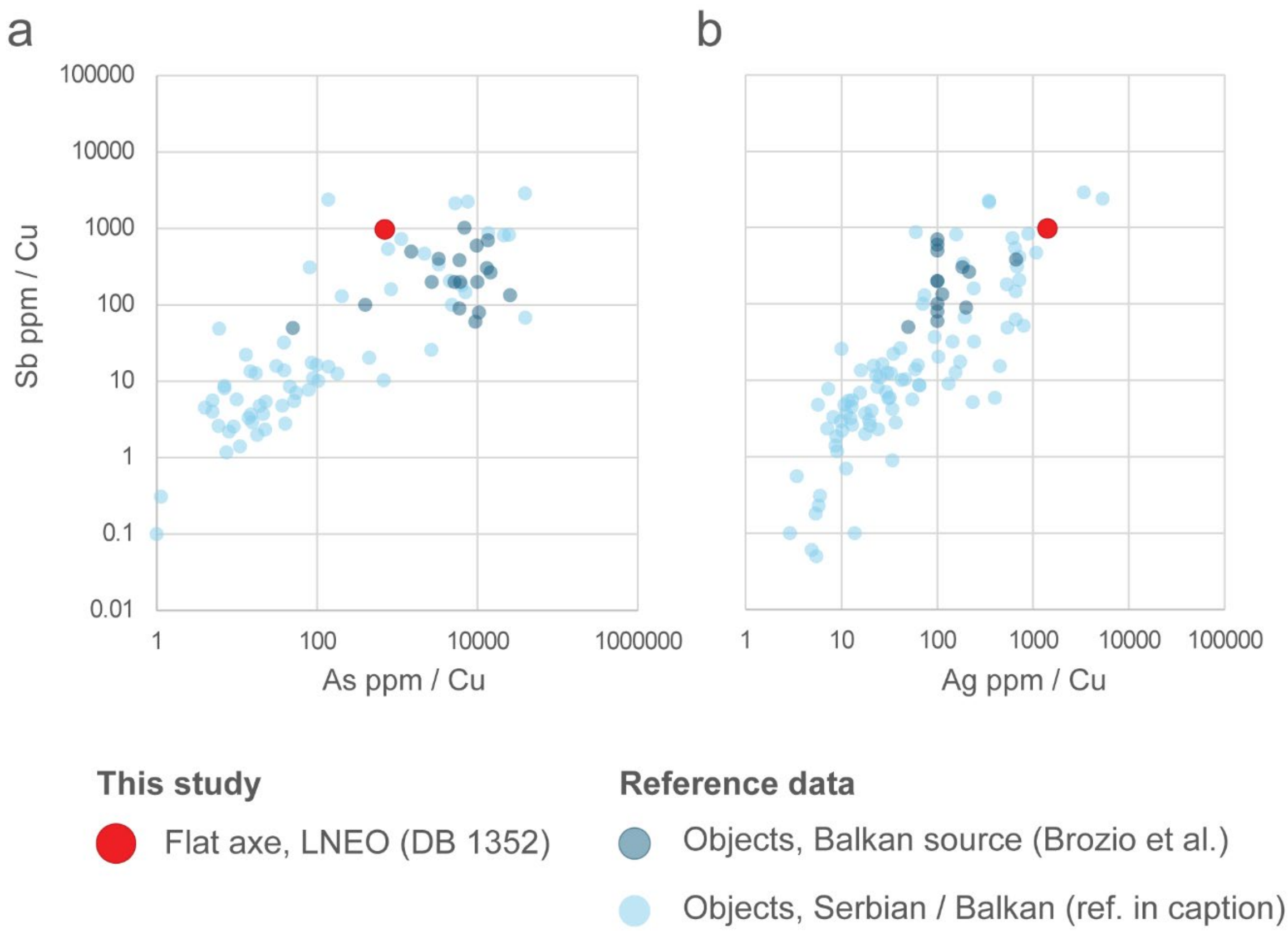

Fig D. a-b) trace elements in Late Neolithic copper flat axe DB 1352 compared to contemporary copper objects from Serbia and the Western [62,64] and objects attributed to Serbian copper sources [65] (diagram: S. W. Merkel).

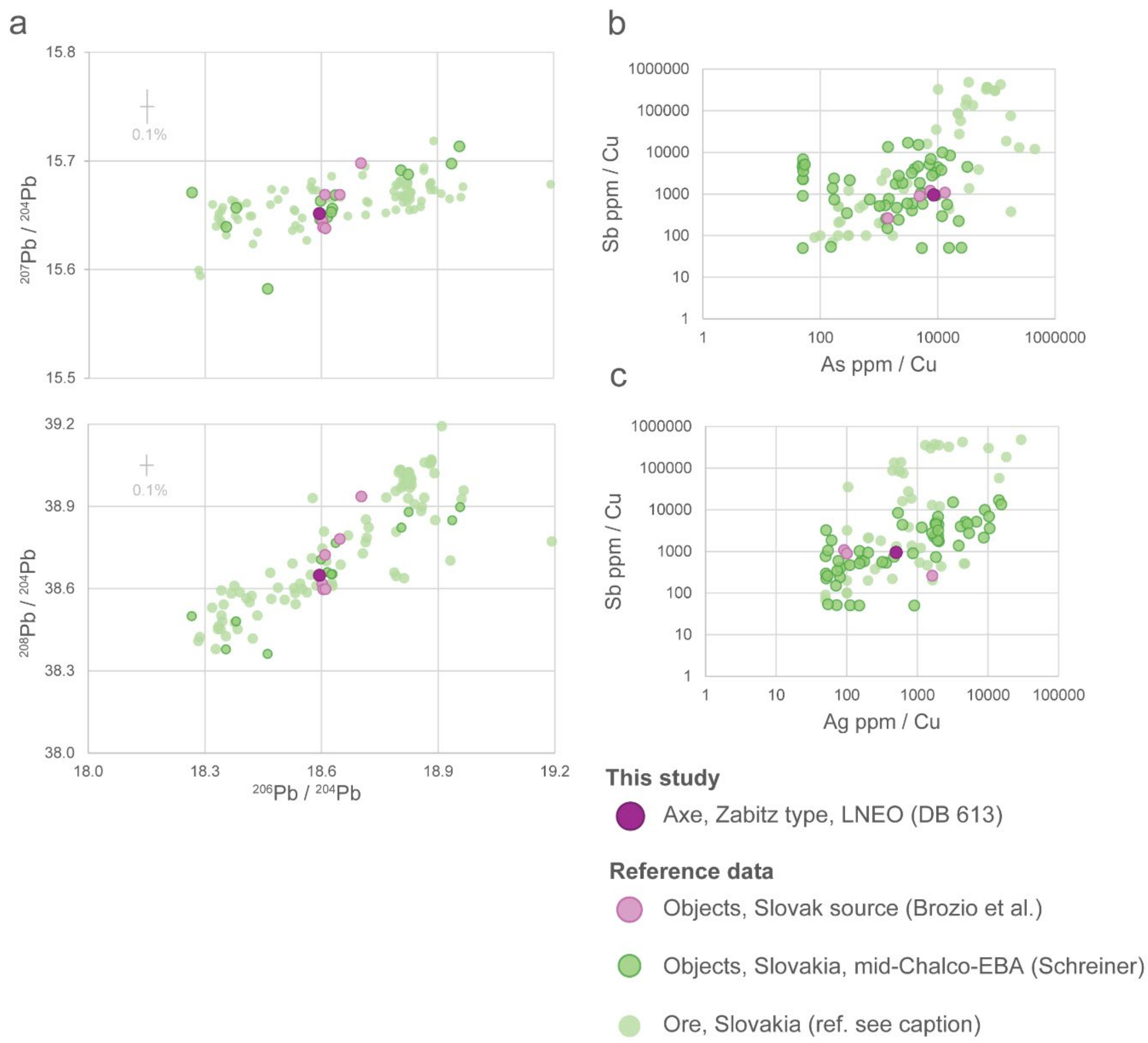

Fig F. a) the lead isotope ratios of a Late Neolithic Zabitz-type double axe (DB 613) compared to Central European object attributed to Slovakian sources [65,69] and Slovakian polymetallic ore [67-69]. Lead isotope ratio errors are expected to be less than 0.1%. b-c) trace element ratios of DB 613 and Slovak ore and metals [65,69] (diagram: S. W. Merkel).

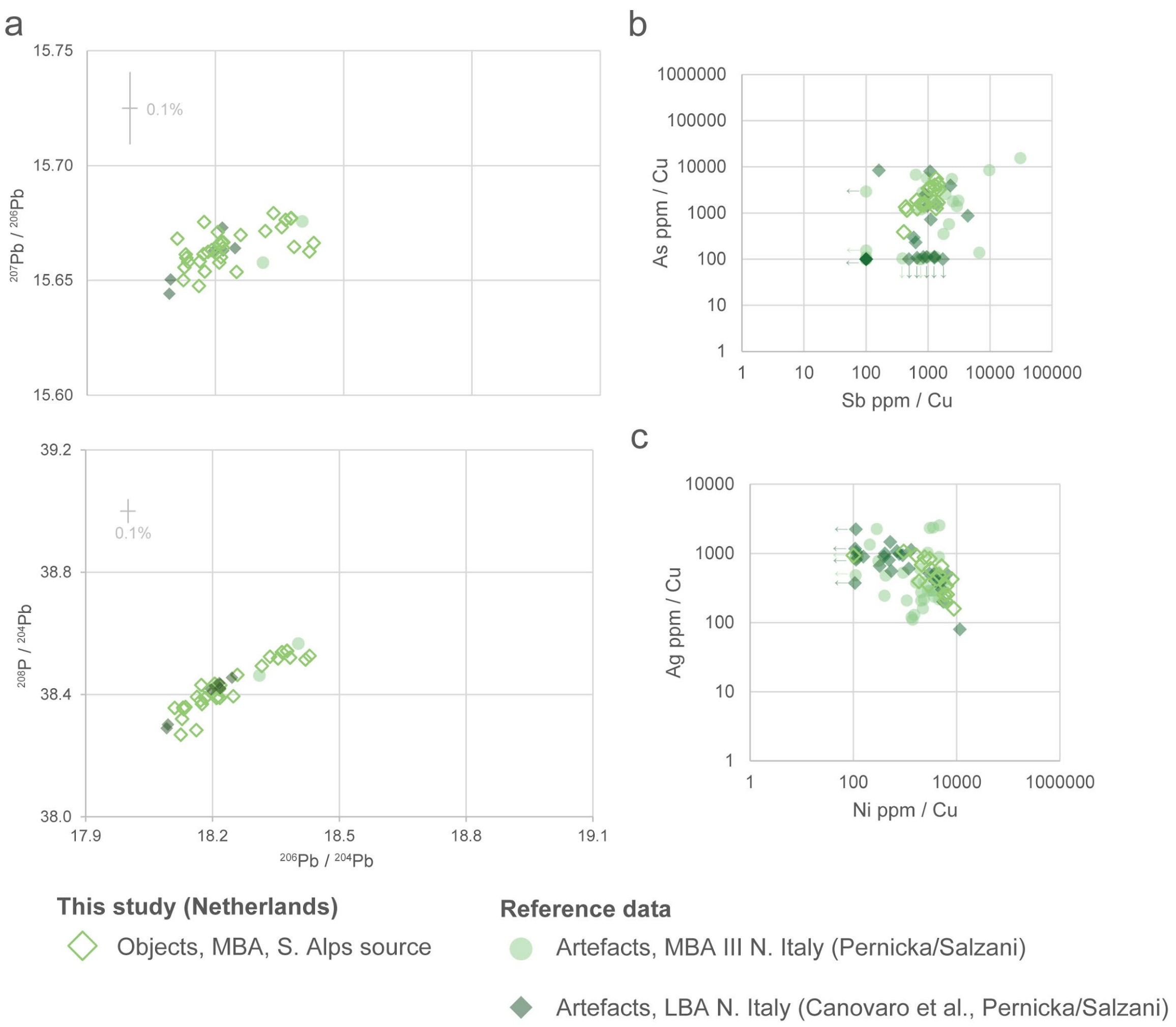

Fig G. a) Lead isotope and b-c) trace element comparison between Dutch-found objects attributed to the South Alpine AATV and Northern Italian MBA/LBA bronze objects [118,119] (diagram: S. W. Merkel).

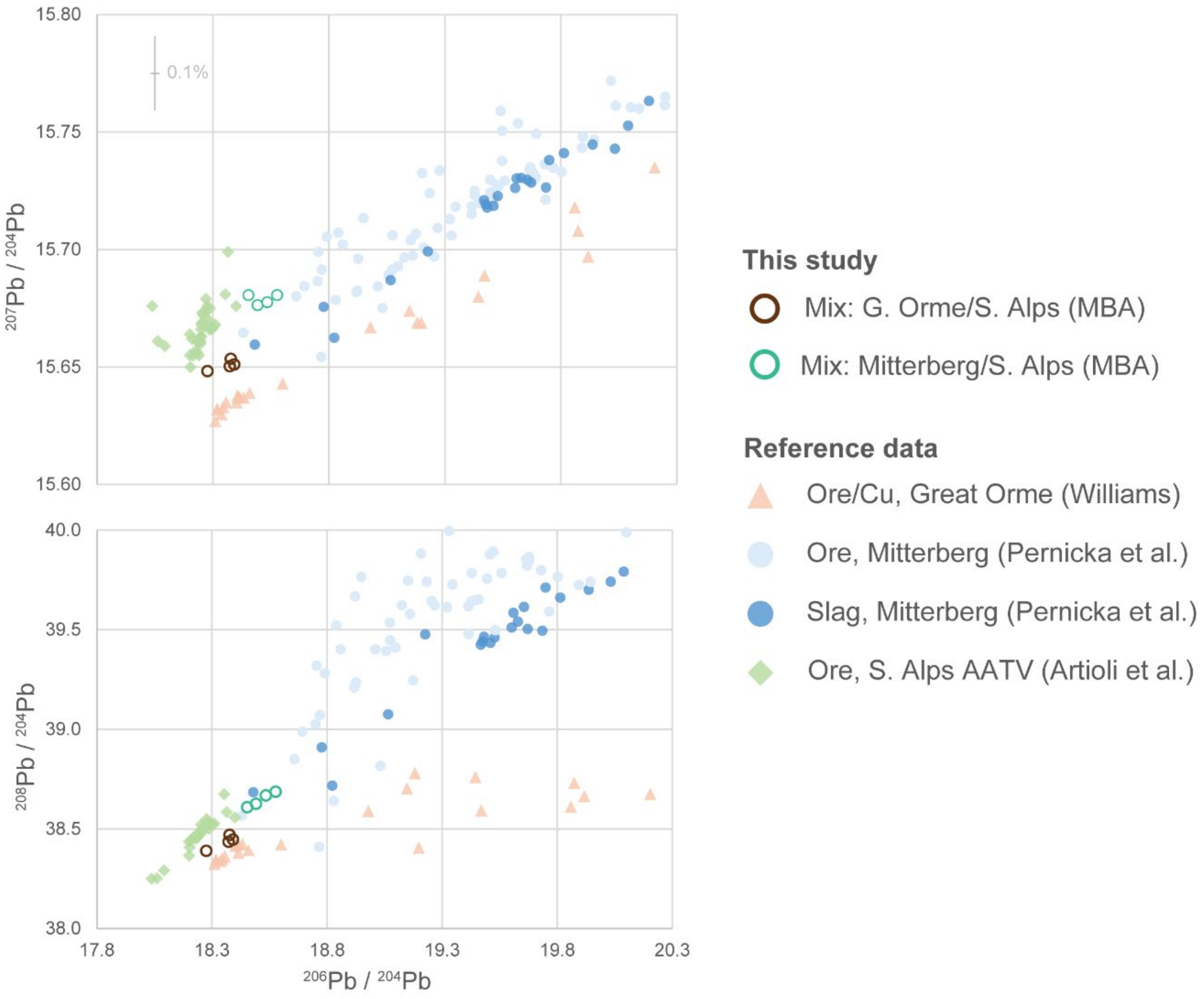

Fig H. Potential mixtures of chalcopyrite-type coppers during the MBA B in the Netherlands. The lead isotope ratios fall between the major MBA chalcopyrite-type sources of the Great Orme [30], Mitterberg [95] and the South Alpine AATV [103] (diagram: S. W. Merkel).

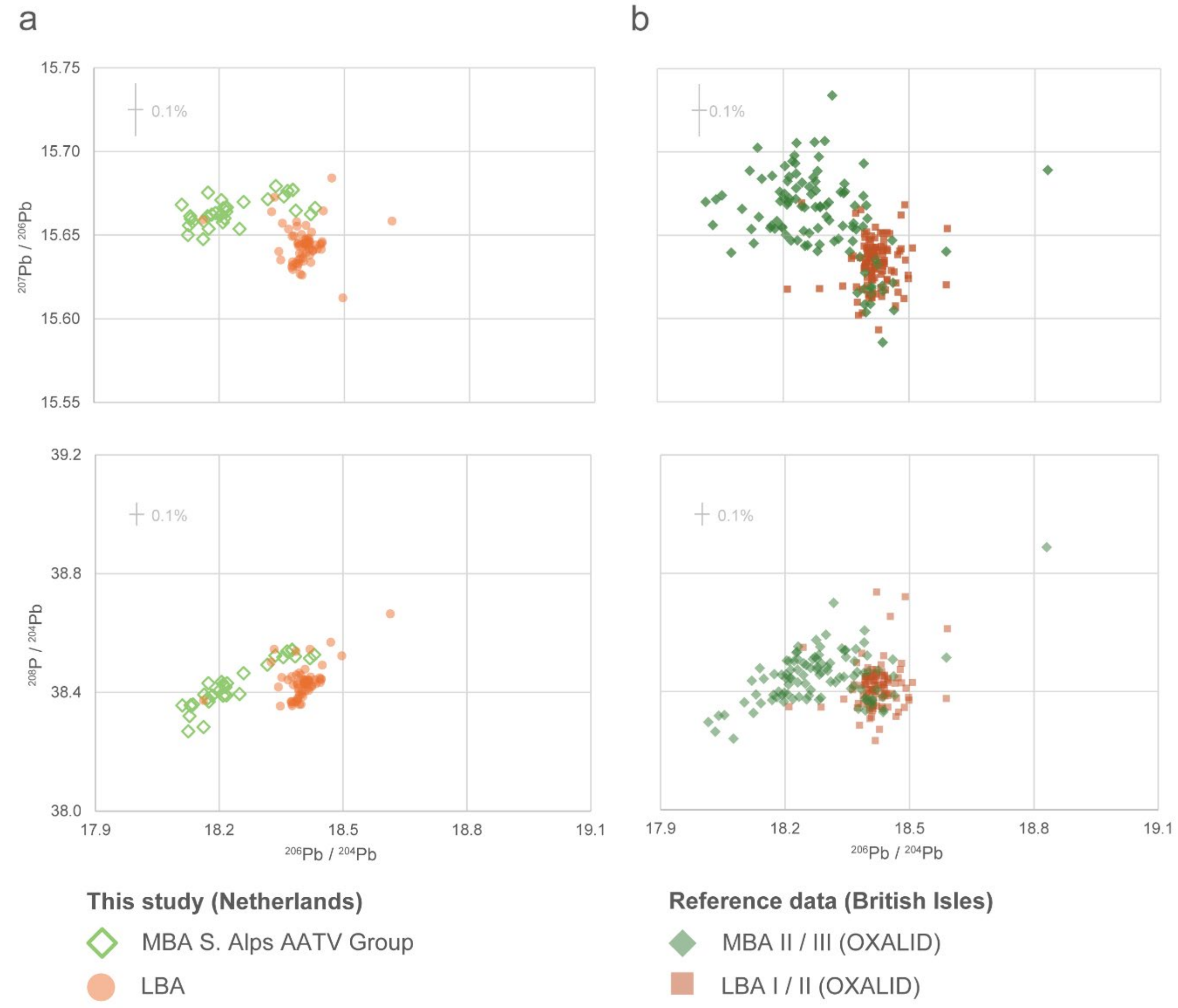

Fig I. Comparison of the lead isotope ratios of a) the MBA South Alpine AATV group from the Netherlands and the LBA and b) MBA II/III and LBA I/II objects from the British Isles [55] (diagram: S. W. Merkel).

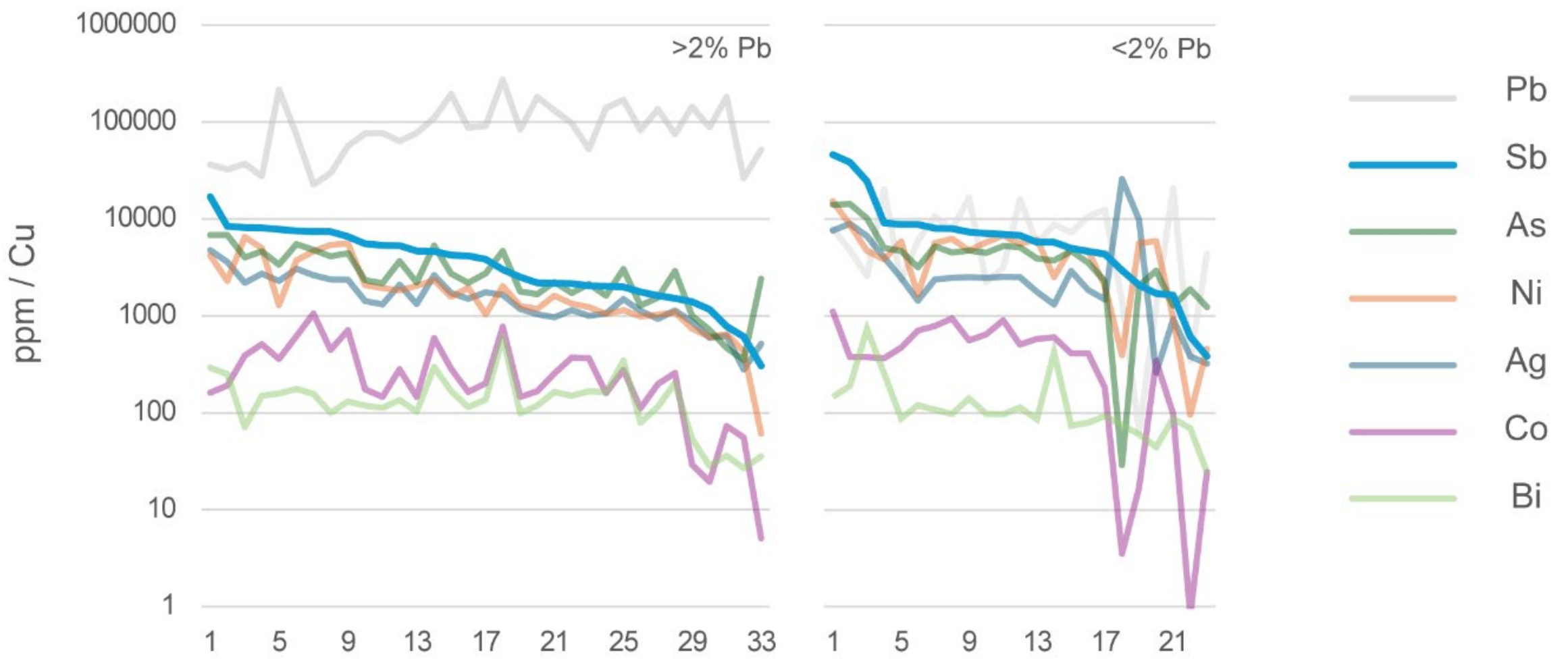

Fig J. Element profiles of leaded bronzes (>2% Pb) and low-poor copper alloys (<2% Pb) found in the Netherlands. The elements are normalized to copper and the copper alloys of each group are sorted by descending Sb content (ICPMS data) (diagram: S. W. Merkel).

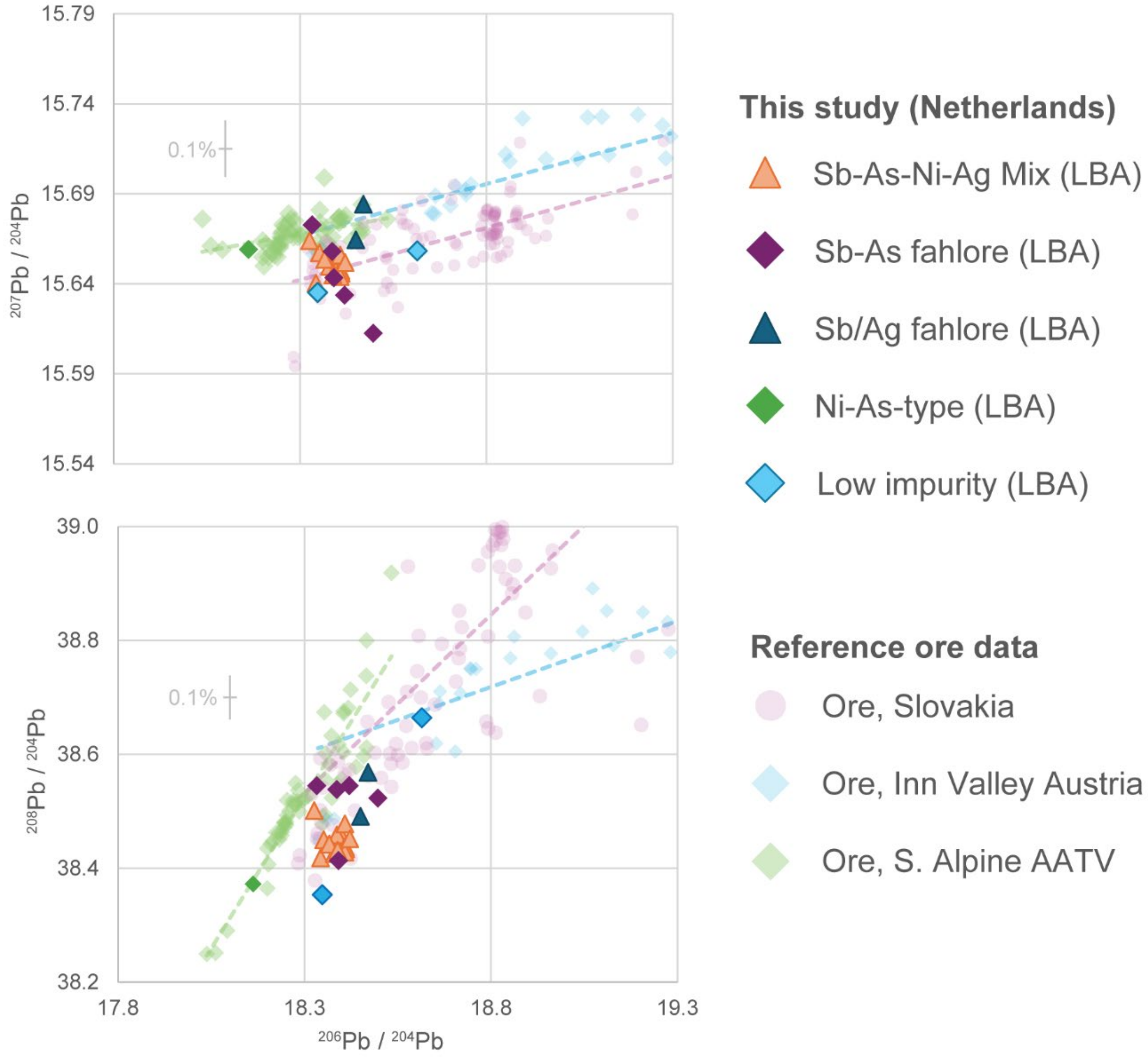

Fig K. Comparison of the lead isotope ratios of diverse LBA alloys from the Netherlands and ore from Slovakia [67-69], the Inn Valley in Austria [124] and the South Alpine AATV [103] (diagram: S. W. Merkel).

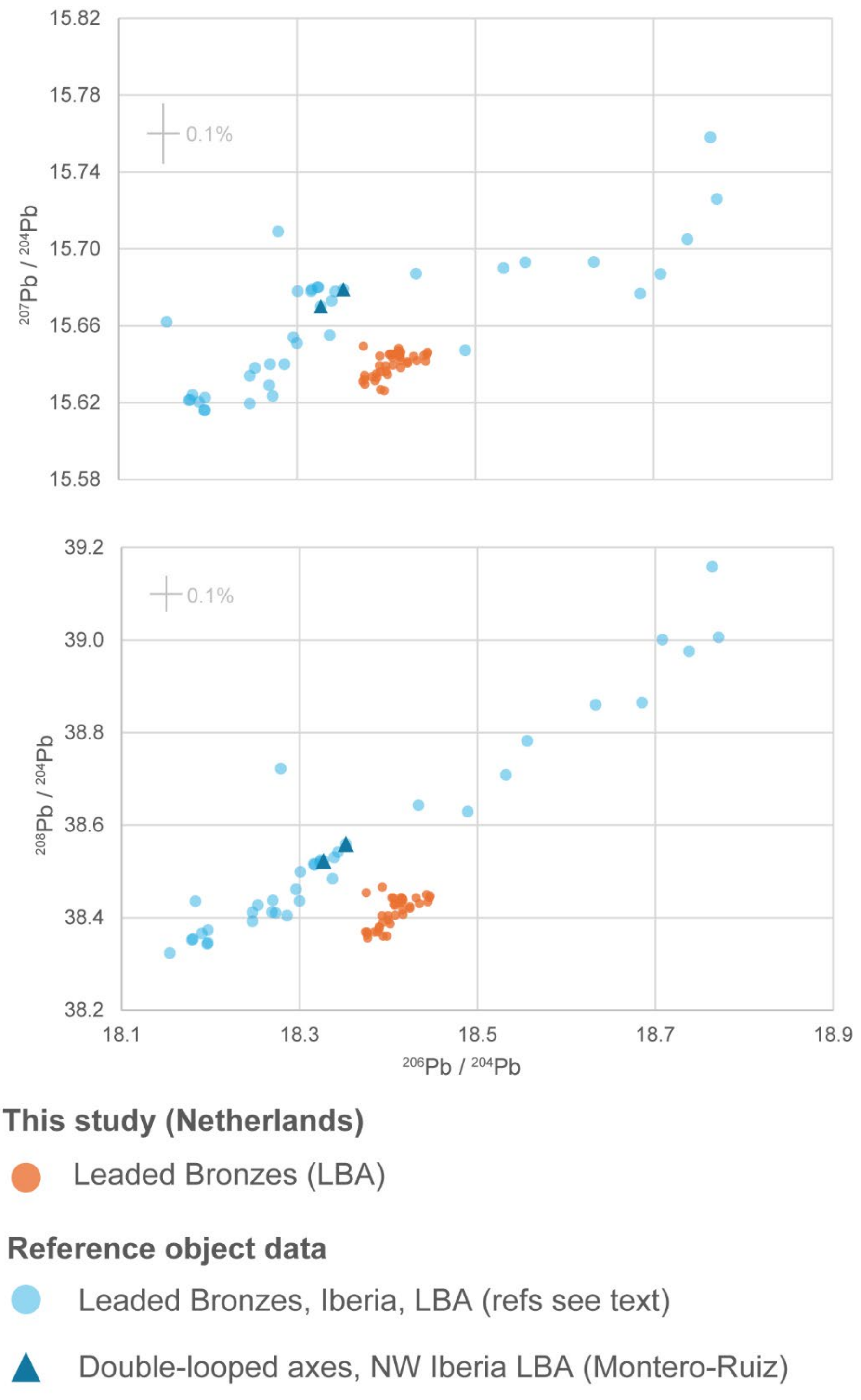

Fig L. Comparison of the lead isotope ratios of leaded bronzes in the Netherlands and Iberian double-looped axe ingots [161] and other leaded bronzes from the Iberian Peninsula [55,166-171,175,177-179] (diagram: S. W. Merkel).
