# Supplementary material for: Tracing Europe-Wide Copper and Bronze flow in the Dutch Late Neolithic to the Early Iron Age
Source: PLoS One. 2026 Sep 8;21(9):e0348751. doi: 10.1371/journal.pone.0348751 (PMC13552965; doi:10.1371/journal.pone.0348751)
Supplement: Supplementary file 2 [file pone.0348751.s002.pdf]

Tracking Europe-Wide Copper and Bronze Flow in the Dutch Late Neolithic to the Early Iron Age

Stephen Merkel, Stijn Arnoldussen, Liesbeth Theunissen, Bertil van Os, Judith van der Leije, Joris Brattinga, Luc Amkreutz, Bibi Beekman, Irini Biezeveld & R. Alan Williams

S2 Appendix. Standards and data quality

Elemental analysis: ICP-QMS

Analytical totals for the standards averaged at 101% and only two analyses (of 35) were beyond ±5% and all were within ±10%. Analytical totals for the samples were within ±10% for approximately two-thirds of the samples. Notably, 22 samples had analytical totals below 80%. There is a slight tendency for samples below 4 mg to have lower analytical totals, possibly as a result of weighing error or loss during sample transfer. This does not account for all low totals: there are weak, but visible, trends that correlate increased Fe (r^2=0.29) and Sn (r^2=0.36) with low analytical totals, which is suggestive of the presence of corrosion products in the sample. Nearly all bronzes with Sn contents above 16 wt. % have analytical totals below 85%, again suggesting the presence of (Sn-enriched) corrosion products. Three samples with totals below 60% are likely to have consisted of corrosion products almost exclusively (DB 2923, DB 2481 and DB 1402).

The method quantification limit (MQL) for trace elements is presented in S3 Table. In terms of defining the lowest limits, the MQL is conservative (10σ / Background Signal), ensuring that the elements detected are quantifiable. The MQL for the elements P (0.125 wt. %), Cr (15 ppm), Se (53 ppm) and Cd (0.3 ppm) are almost always higher than the concentrations measured in the samples, but for all other elements, sample concentrations were regularly above the MQL.

Correlation between the ICP-QMS measurements of copper-alloy reference materials and their certified values are in all cases greater than 0.99 r^2 (Fig A in this supplement). The accuracy for the seven reference copper alloys is 100 ±2% (Cu), ±5% (Ni, As, Zn, Cd, Au), ±10% (Pb, Sn, Ag, Sb, P, Fe, Co, Bi) and ±20% (Cr and Mn). Average precision of five repetitions of the seven standards (2RSD) is <0.5% (Cu), <2% (Sn, Co, Bi), <5% (Pb, Ag, Ni, As, Sb, Zn), <10% (Cd), <20% (Mn, Fe, Au) and >20% (P and Cr).

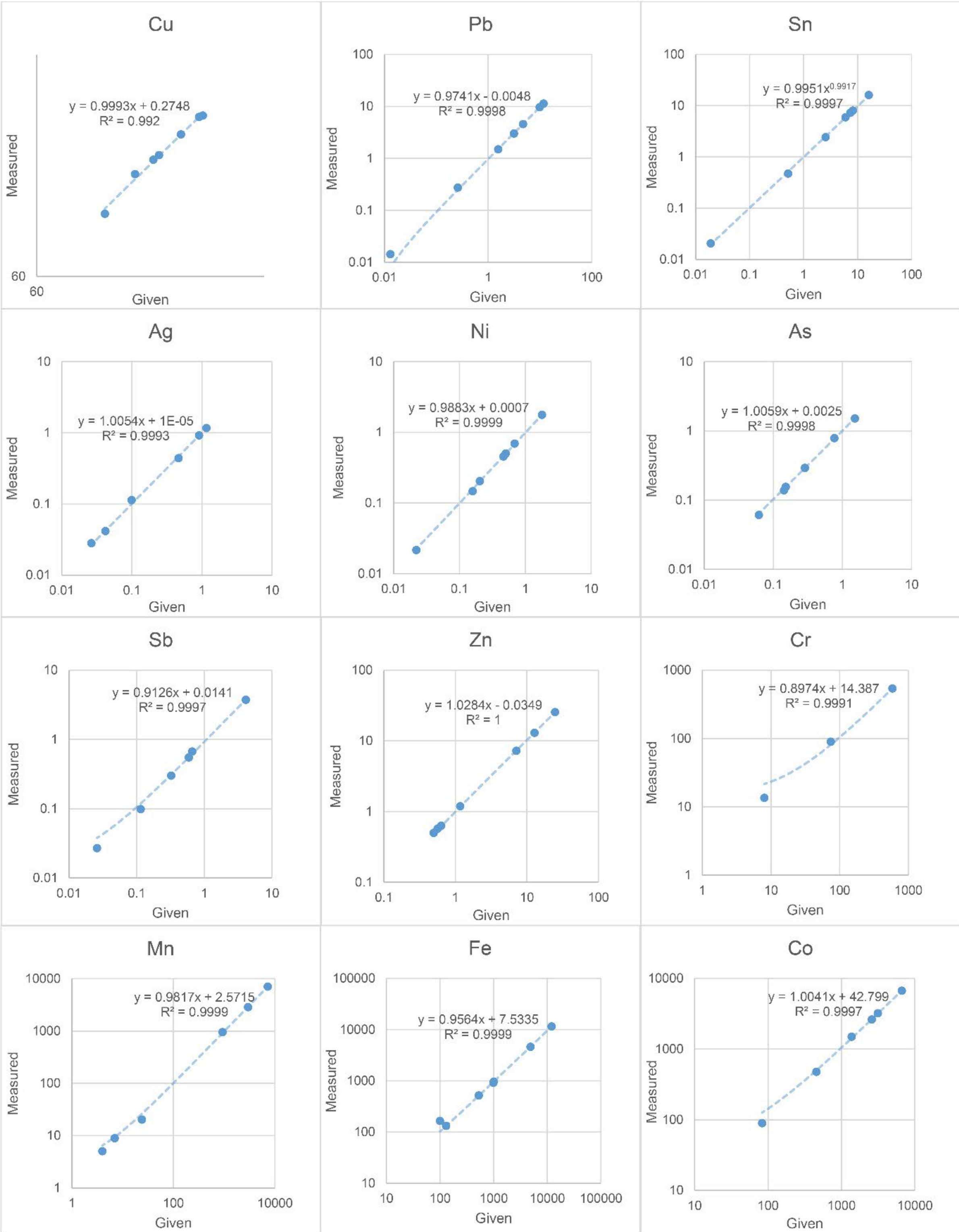

Fig A. Averages of ICP-QMS analyses of copper alloy standards compared to given certified values.

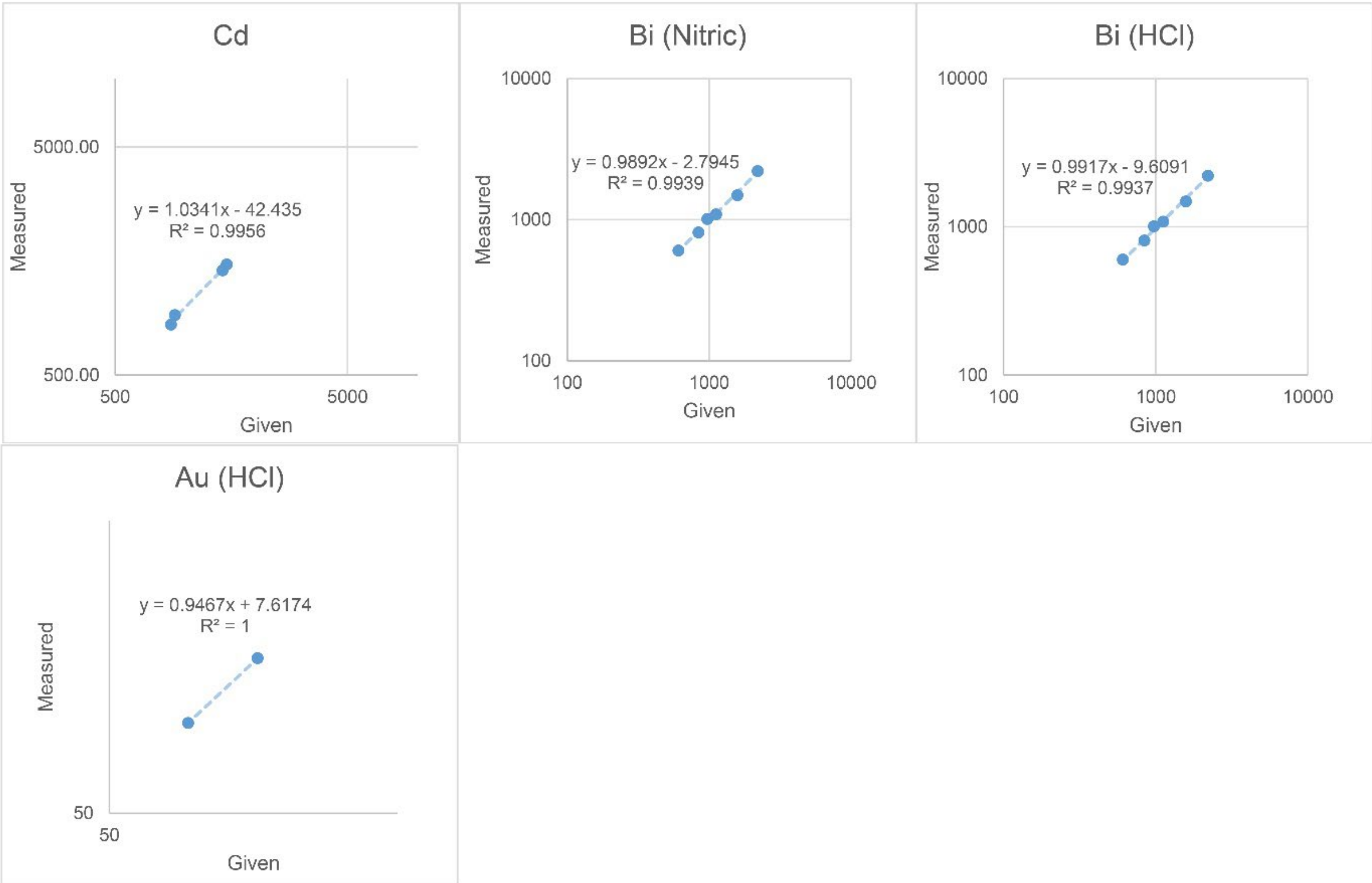

Fig A. continued.

Portable X-ray fluorescence vs ICP-QMS

All objects were measured using pXRF on the surface and, when destructive sampling was permitted, also on the drilled sample material. Because of corrosion effects, non-destructive surface elemental analyses of copper-based alloys can be problematic (Fernandes et al. 2013; Orfanou and Rehren 2015). Efforts were made to reduce the effects of corrosion by through repeat measurements and by seeking areas with the least patena. The surface enrichment of lead and tin appear to be the key issues.

The analyses of prepared surfaces under controlled conditions (i.e. the use of a docked pXRF with lead-lined analysis chamber) can be better assessed. Analyses of the CHARM are presented in Roxburgh et al. (2019). In our current study, we present the pXRF analyses of drillings which can be directly compared to the ICP-QMS analyses of the same sample (Figure B in this supplement). Lead has one of the highest correlations (> 0.9 r^2), but arsenic, nickel, silver and antimony are generally strongly correlated when above the detection limit of the pXRF.

The data collected in the present study allows for the systematic comparison of surface pXRF and the pXRF and ICP-QMS analysis of drilled samples. This comparison is out of the scope of the present study and is the focus of a separate paper in preparation (van Os et al. in prep.). The surface pXRF results are presented here as qualitative data, in which the accuracy and precision cannot be specified with certainty for each individual object.

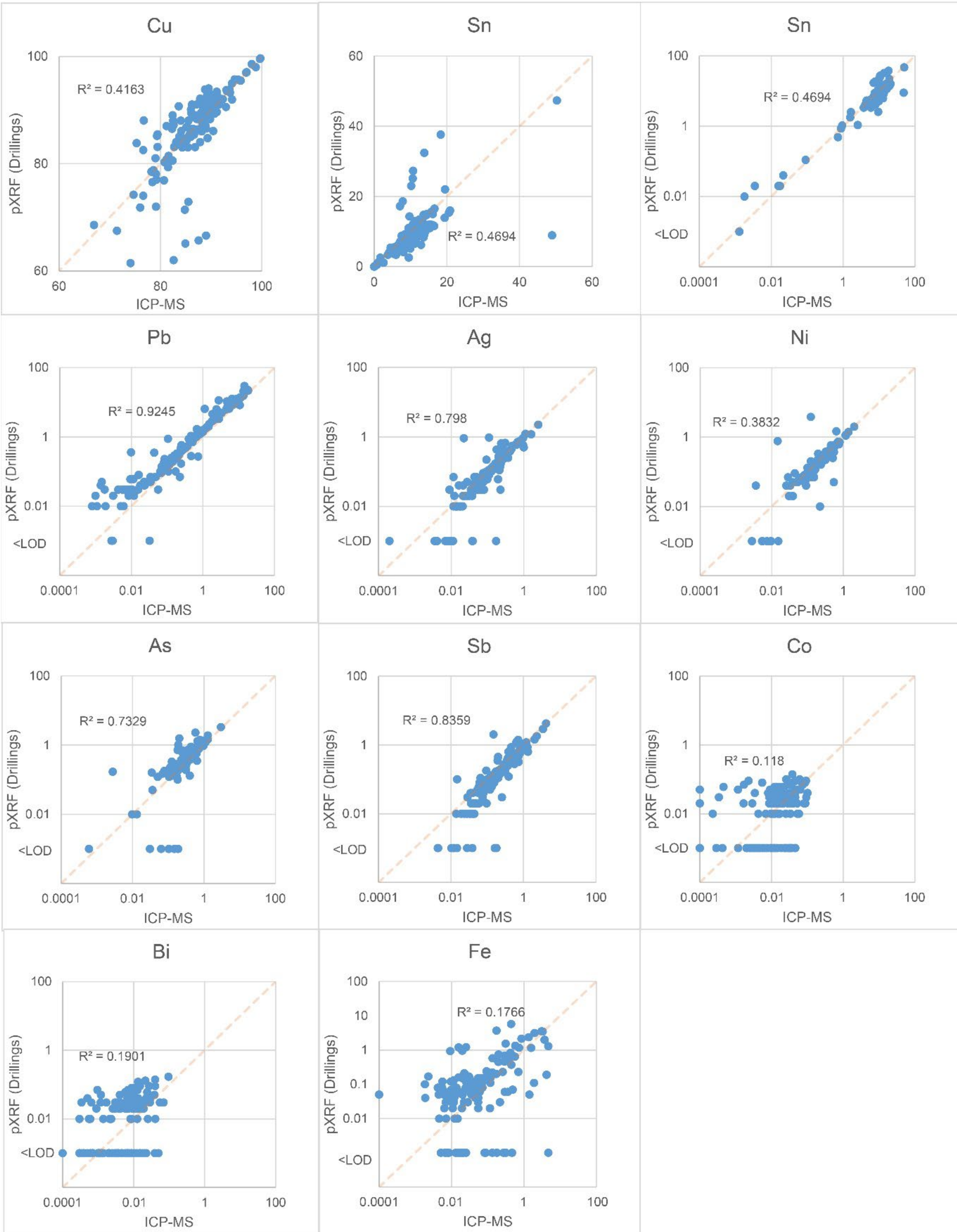

Fig B. Method comparison between ICP-QMS and pXRF of drilled samples of the same artefacts. The dotted orange line represents the ratio of 1:1.

Lead isotope analysis

The samples were measured over a 16-month period in 12 sessions. The measurements of CPI are all accurate within the long-term values for the instrument. The VU long term precision (2SD) of CPI is ±0.003 for $^{206}Pb/^{204}Pb$ and $^{207}Pb/^{204}Pb$ and ±0.011 for $^{208}Pb/^{204}Pb$, which is the best proxy for the analytical precision. In one session, signal drift seriously impacted the precision of the measurements. All affected samples were re-analyzed under stable conditions, but two low-lead samples could not be repeated: DB 193 and DB 205. These two samples are expected to have four-times higher uncertainties than the long-term precision.

The seven portable laser ablation full-method blanks were typically 95 pg (range 17 pg to 264; not taking into account one outlier at 506 pg). The eight bulk full-method blanks averaged at 83 picograms (range 18 pg to 233 pg). These are negligible compared to the amount of lead processed (lead recoveries: 2/3 of samples 500-2000 ng, 1/6 between 100-500 ng, 1/6 less than 100 ng). The eight samples with the lowest lead analyzed (10-20 ng) are pLA samples collected at the RMO (marked in data table as measured at 10 ppb). The associated blanks from the RMO were 82 pg, which is more than 100-200 times lower than the smallest lead samples measured.

The portable laser ablation sampling for lead isotope analysis was successful in 54 of 55 cases. The failure of one sample was due to an anomalously high and unrepresentative surface lead reading by pXRF which led to a gross overestimation of the lead content of the metal. As a result, too few ablations were carried out to ensure a robust measurement of ^{204}Pb and the sample was disregarded.

The pLA test shows that the two pLA samples and independently drilled/measured duplicates are within the long-term precision of the instrument.

References

Fernandes R, van Os BJH, Huisman DJ.2013, The use of hand-held XRF for investigating the composition and corrosion of Roman copper-alloyed artefacts, Heritage Science. 2013;1:30.

Orfanou V, Rehren Th. A (not so) dangerous method: pXRF vs. EPMA-WDS analyses of copper-based artefacts. Archaeological and Anthropological Sciences. 2015;7:387-397.

Van Os B, Arnoldussen S, Theunissen L, Merkel S. Seventy years of analyzing archaeological bronzes from Netherlands. A comparison of methods and best practices for answering archaeological questions with analytical tools. (in prep.).

Roxburgh MA, Heeren S, Huisman DJ, van Os BJH. Non-destructive survey of early Roman copper-alloy brooches using portable X-ray fluorescence spectrometry. Archaeometry. 2019;61(1):55-69.
